# Aberrant skeletal muscle morphogenesis and myofiber differentiation characterize equine myotonic dystrophy

**DOI:** 10.1371/journal.pone.0341655

**Published:** 2026-01-29

**Authors:** Stephanie J. Valberg, Zoë J. Williams, Elizabeth G. Ames, James R. Mickelson, Yvette S. Nout-Lomas, Gabriele Landolt, Macarena Sanz, Keri Gardner

**Affiliations:** 1 Michigan State University, Large Animal Clinical Sciences, College of Veterinary Medicine, East Lansing, Michigan, United States of America; 2 Department of Pediatrics, Michigan Medicine, University of Michigan, Ann Arbor, Michigan, United States of America; 3 Department of Pathobiology, College of Veterinary Medicine, University of Minnesota, St Paul, Minnesota, United States of America; 4 Department of Veterinary Clinical Sciences, Washington State University, Pullman Washington, United States of America; UCL: University College London, UNITED KINGDOM OF GREAT BRITAIN AND NORTHERN IRELAND

## Abstract

Equine myotonic dystrophy (eMD) is a rare neuromuscular disorder of undetermined origin marked by muscle hypertrophy and stiffness, dystrophic muscle histopathology, and myotonic discharges. In humans, myotonic dystrophy (DM) arises from trinucleotide repeat expansions in dystrophia myotonica protein kinase (*DMPK*) (DM1) or tetranucleotide expansions in cellular nucleic acid-binding protein (*CNBP*) (DM2), which disrupt mRNA processing and induce embryonic splicing patterns across multiple genes. In 6 eMD Quarter Horse types, (2–36 months-of-age) and 8 control Quarter Horses we determined: (1) fiber type composition of triceps*,* gluteal, and semimembranosus muscles; (2) differential gene (DEG) and protein (DEP) expression using transcriptomic and proteomic analyses; (3) presence of repeat expansions in transcripts of *DMPK* or *CNBP* and (4) exon 7 retention in *CLCN1* or exon 22 splicing in *ATP2A1*. Predominance and clustering of type 1 fibers, expression of embryonic myosin, and upregulated mitochondrial and sarcomeric DEPs characterized eMD hindlimb musculature. Gene ontology (GO) analysis of 730 upregulated DEGs identified numerous GO terms related to morphogenesis of mesoderm-derived tissues and upregulated genes impacting myoD expression in eMD muscle. Top upregulated DEG involved myogenesis (*MYOZ2, SBK2, SBK3, PAMR1*), neurons, transcription/translation, cytoskeleton, basement/plasma membranes, and calcium binding/transport. Top upregulated proteins also impacted muscle morphogenesis (MUSTN1, CSRP3, TMSBX4, PDLIM, CALD1) as well as categories of mitochondria, sarcomere, extracellular matrix/ basement membrane, transcription, translation, cell cycle regulation, neurons amongst others. Downregulated DEP primarily impacted mitochondria, the sarcomere and glycogen metabolism. Notably, unlike human myotonic dystrophy, trinucleotide repeat expansions were not found in the *DMPK* 3’UTR (CTG)_n_ nor tetranucleotide repeat expansions (CCTG)_n_ in intron 1 of *CNBP*. Isoforms of *CLCN1* containing fetal exon 7 were detected in equal frequency in eMD and control muscle and exon 22 was not alternatively spliced in *ATP2A1* as has been found in DM1. Thus, distinct from DM1 and DM2, eMD is driven by unique molecular mechanisms impacting skeletal muscle morphogenesis, neurons and regulation of gene transcription/translation that alter fiber type composition, distribution and morphology. The origin of myotonia does not appear to be driven by a mutation in *CLCN1* or retention of exon *CLCN* 7. Expanded splice site analysis and further research is warranted to elucidate the cause of myotonia and the distinct etiology of eMD.

## Introduction

Two forms of myotonia, myotonia congenita and myotonia dystrophica are recognized in horses. [1] Both forms are characterized by high frequency waxing and waning myotonic discharges in electromyography (EMG) of skeletal muscle. [1,2] Myotonia congenita presents with normal muscle histology, whereas myotonia dystrophica is marked by distinctive dystrophic skeletal muscle histopathology. [1] Both conditions are rare in horses with only 9 equine myotonic dystrophy (eMD) cases documented in the literature and no information published on inheritance. [3–10] Clinical signs begin within a few months of birth and include pronounced muscle development predominantly in the hindquarters, stiffness while ambulating, and visible prolonged muscle contractions following tactile stimulation. Two cases report testicular atrophy/hypoplasia cataracts and one case mild lenticular opacities. [4,9] Muscle biopsies of eMD horses are characterized by muscle fiber size variation, internalized myonuclei, sarcoplasmic masses and muscle fiber type grouping [3–10].

In humans, the cause of myotonic dystrophy (DM) is well known, a dominant toxic RNA gain-of-function arising from tri or tetra nucleotide repeats. [11] Type 1 myotonic dystrophy (DM1) is due to an unstable expansion of (CTG)_n_ repeats in the 3’ untranslated region (UTR) of the dystrophia myotonica protein kinase (*DMPK*) gene. In type 2 myotonic dystrophy (DM2) there is an unstable (CCTG)_n_ expansion in the first intron of cellular nucleic acid binding protein (*CNBP*). [11] These large repeat expansions are fully transcribed and polyadenylated resulting in the *DMPK* or *CNBP* transcripts being trapped in the nucleus and sequestering muscleblind-like (MBNL) protein, which is crucial for normal splicing of many pre-mRNAs. [11–13] Additionally, CELF1 protein has elevated steady-state levels in DM1 skeletal muscle and it promotes fetal or neonatal splice isoform. [11] As a result, a transition from fetal to adult splicing patterns arises during adulthood resulting in numerous proteins in adult DM patients having aberrant fetal splicing patterns. [12–14] These include the chloride channel (CLCN1) which causes myotonic discharges, the sarco-endoplasmic reticulum calcium ATPase 1 (SERCA1) among others and expression of embryonic myosin heavy chain (MYH3) in adult muscle [12–14].

The purpose of the present study was to: (1) Characterize the histopathology of triceps*,* gluteal, and semimembranosus muscles in eMD horses; (2) Compare muscle fiber type composition and embryonic myosin expression between eMD and control horse; (3) Evaluate differential gene and protein expression in eMD versus control horses muscle; (4) determine if repeat expansions are present in *DMPK* or *CNBP* gene transcripts, and (5) Determine if, similar to DM1, alternative splicing patterns of *CLCN1* and *SERCA1* exist in in eMD horses.

## Results

### Histopathology

#### Control horses.

**Fig 1:**
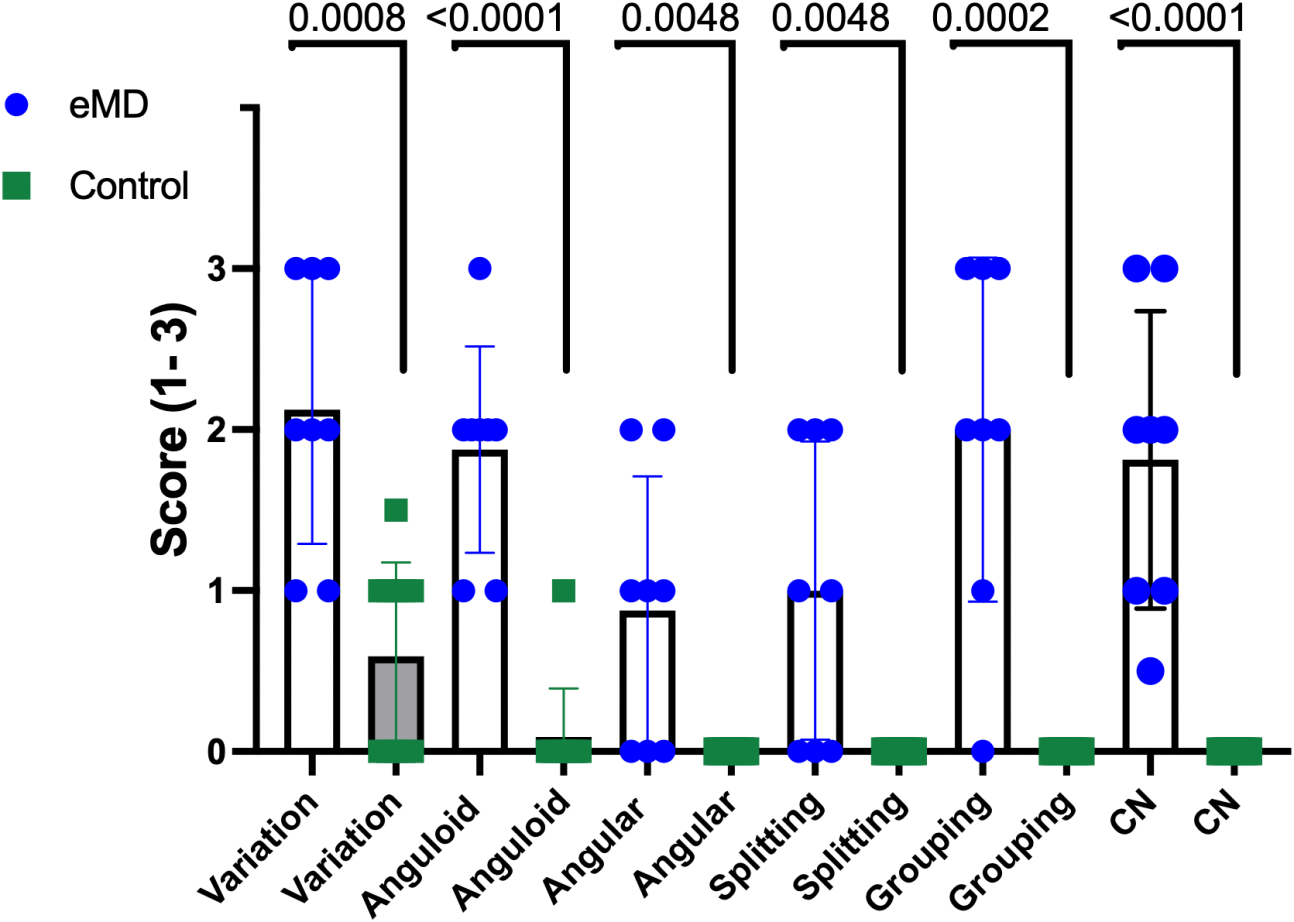
Scores for muscle fiber size variation, anguloid and angular atrophy, fiber splitting, fiber type grouping and centrally displaced myonuclei in gluteal or semimembranosus muscle of 8 horses with myotonic dystrophy (eMD) and 9 controls. Scoring system was 0 = not present, 1 = mild alterations were present in<20% of 10x field, 2 = moderate alterations present in 21–50% of 10X field and 3 = severe alterations present in > 50% of 10X fields. P values are shown based on statistical comparisons performed with a Mann Whitney test.

No histopathologic abnormalities were noted in the gluteal and semimembranosus muscle samples of 7/9 control horses (Fig 1, S1 Table). Mild anguloid atrophy and a few blood vessels with mononuclear cuffing were present in semimembranosus samples from 2 control horses that were used for fiber typing.

#### eMD Horses.

**Fig 2:**
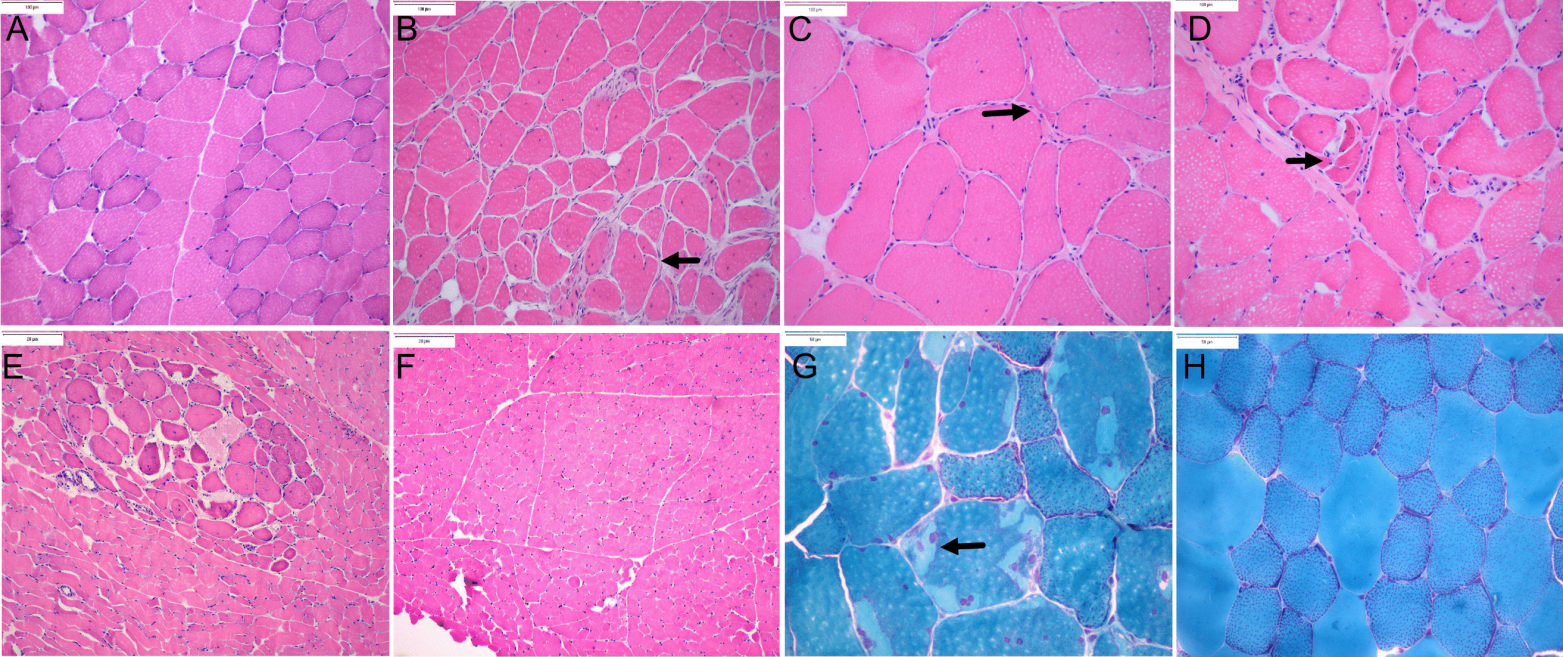
Muscle histopathology in frozen sections of eMD horses. **(A)** Normal HE stain of the gluteal muscle of a 4-year-old control horse (C2). **(B)** Marked fiber size variation, anguloid atrophy, fiber splitting (arrow) internalized myonuclei and increased endomysial connective tissue in semimembranosus muscle of 2-month-old eMD1 (HE 20X). **(C)** Large myofibers and anguloid and angular (arrow) atrophied fibers, fiber splitting and internalized nuclei in gluteal muscle of 3-year-old eMD3 (HE 20X). **(D)** Marked variation in fiber sizes, anguloid and angular atrophy (arrow) and internalized nuclei in 3-month-old eMD2 (HE 40X) **(E)** Areas of marked fiber size variation with a focal area of larger fibers juxtaposed with atrophied fibers in gluteal muscle of 3-month-old eMD2 (HE 20X). **(F)** Normal appearing triceps muscle in eMD2 (HE 10X) **(G)** Internalized myonuclei and sarcoplasmic masses (arrow) in 3-year-old eMD5 (modified Gomori Trichrome 40X). **(H)** Normal gluteal muscle of 3-year-old control horse C2 (modified Gomori Trichrome 40X).

The signalment and clinical signs of eMD horses are shown in Table 1. Compared to control horses, the gluteal and semimembranosus muscles of eMD horses had significantly greater variation in muscle fiber sizes, anguloid atrophy, angular atrophy, and single or multiple internalized myonuclei (Figs 1, 2a-e, S1 Table). Epimysial fibrosis (Fig 2b-d), fiber splitting (Fig 2d), mild myodegeneration, ringbinden fibers and sarcoplasmic masses (Fig 2g) were also present in many eMD horses but not controls (S1 Table). The triceps muscle of 2/4 eMD horses appeared normal (Fig 2f) whereas in the other 2 eMD horses anguloid atrophied fibers were evident and in one horse (eMD6) some triceps fascicles had focal clusters with fiber size variation, including larger fibers and angular atrophied fibers and many fibers with internalized myonuclei (S1Table).

**Table 1:** Signalments and phenotype of the horses which presented with myotonia. AP= Appaloosa, QH = Quarter Horse.

Horse	Breed	Age (months)	Sex	Clinical signs
eMD1	AP	2	male	Large hindquarter muscle mass, progressive severe stiffness
eMD2	QH	3	male	Large hindquarter muscle mass, progressive severe stiffness
eMD3	QH	36	female	Large hindquarter muscle mass, severe muscle spasms
eMD4	Paint	24	female	Small stature, stiff gait, and intermittent marked spasms
eMD5	AP	36	castrated male	Small stature, progressive stiffness and spasms
eMD6	AP	36	castrated male	Progressive stiffness, spasms, focal hypertrophy and atrophy
eMD7	QH	1	male	Large hindquarter muscle mass, progressive severe stiffness
eMD8	QH	2	male	Large hindquarter muscle mass, progressive severe stiffness

**Fig 3:**
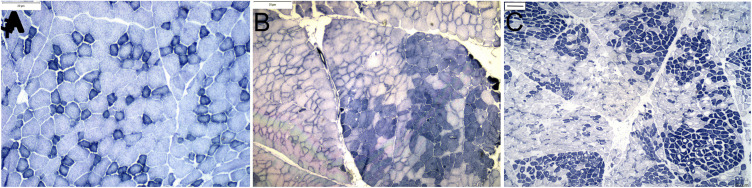
Oxidative fiber typing. (A) Mosaic distribution of oxidative fibers in gluteal muscle from 5-year-old control horse C1(10X). **(B)** Oxidative fiber type grouping in the semimembranosus muscle of 3-year-old eMD3 (NADH-TR 10X) **(C)** Grouped oxidative fibers in gluteal muscle of 3-month-old eMD 2 (NADH-TR 4X). (bar = 20 μm).

Compared to the mosaic fiber type distribution in controls (Fig 3a), marked oxidative fiber type grouping was evident in eMD gluteus and semimembranosus muscles (Fig 3b,c). In some regions of eMD hindlimb muscles and in one eMD triceps muscle (eMD6), grouped oxidative fibers were larger than nonoxidative fibers within the same sample contrasting controls. (Fig 3a-c).

### Muscle fiber types

**Fig 4:**
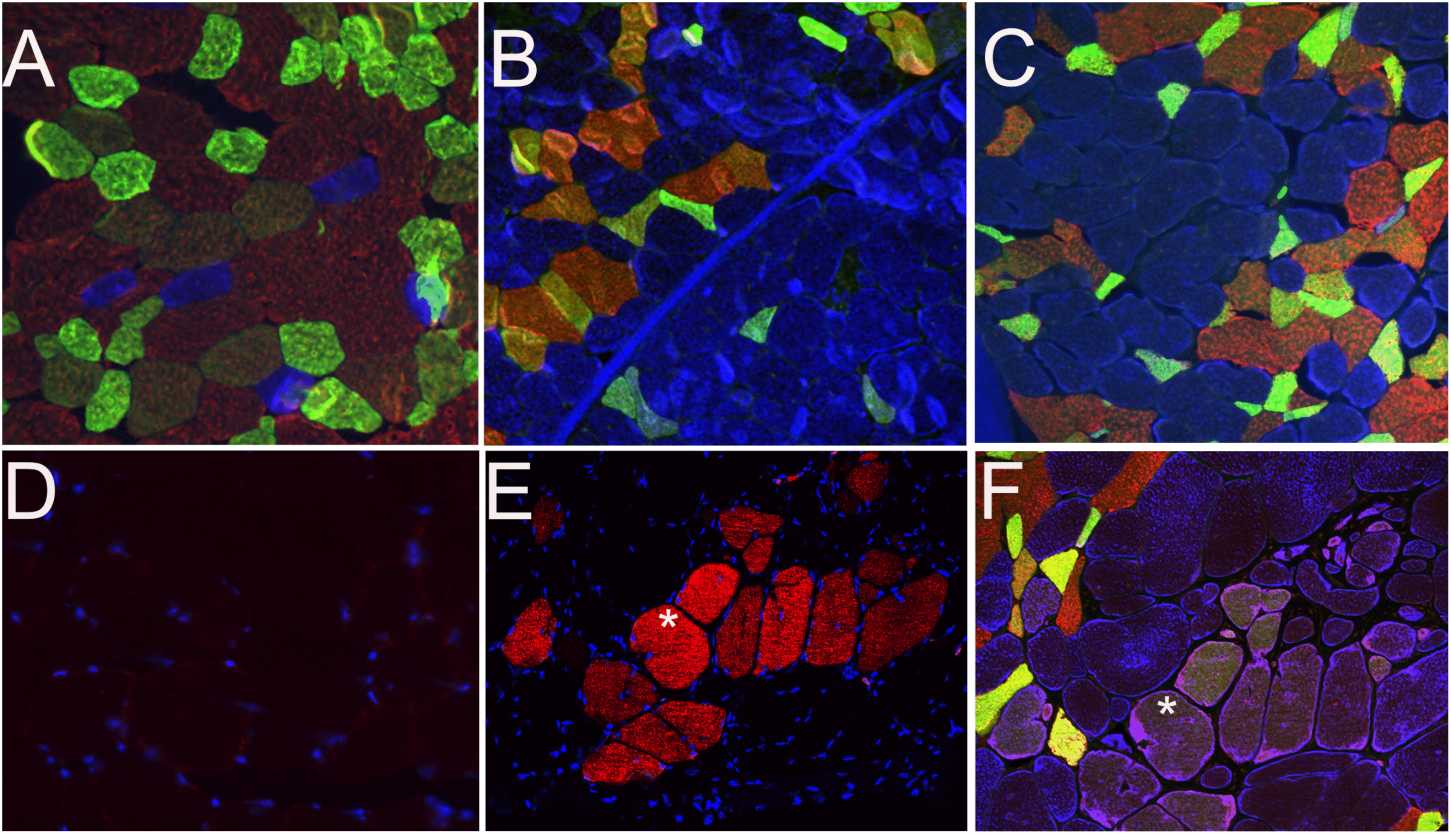
Immunofluorescent fiber typing. **(A)** The mosaic distribution of type 1 (blue) type 2A (green) and type 2X (brown) fibers in a control horse **C1.** Note that type 1 fibers are smaller relative to type 2 fibers. **(B)** Grouping of type 1 fibers (blue) in the semimembranosus muscle of eMD2. **(C)** Grouping of type 1 fibers in semimembranosus muscle of eMD1. Note that type 1 fibers are larger relative to type 2 fibers. **(D)** Staining for developmental myosin (MYH3) in gluteal muscle of control horse C3 with no MYH3 fibers identified. **(E)** Presence of developmental myosin in semimembranosus muscle fibers of eMD2. **F.** Serial section of eMD2 showing that developmental fibers in E stain correspond to type 1 fibers in the type 1, 2A, 2X fiber typing.

Marked contractile fiber type grouping was apparent in gluteal and semimembranosus muscles of all eMD horses contrasting the controls (Figs 4a,b,c, S1 Fig). Compared to controls, eMD gluteal and semimembranosus muscles had significantly more type 1 fibers (6 times more) (eMD mean 42 ± 17%, controls 9 ± 4%, *P* = 0.004) and significantly fewer type 2X fibers (eMD mean 28 ± 10%, controls 55 ± 17, P = 0.03) (Figs 4a,b,c, S1, S2 Fig). Subjectively, in eMD horses, the diameters of many type 1 fibers appeared larger than those of type 2 fibers in regions with fiber type grouping, whereas type 1 fibers appeared smaller than type 2A and 2X fibers in controls (Figs 4a,b,c, S1 Fig).

#### Embryonic myosin.

Fetal muscle from the positive control stained darkly for embryonic myosin (MYH3) (S3 Fig). Hindquarter muscle from control horses and triceps muscle from eMD horses and had no staining for MYH3 (Figs 4d, S3 Fig). In contrast, semimembranosus and gluteal muscles from 4/6 eMD horse were positive for MYH3 fibers (eMD1 23 fibers, EMD2 30 fibers, eMD4 9 fibers, eMD 5 6 fibers per section) (Figs 4e, S3 Fig). Fibers expressing embryonic myosin appeared to have been typed as type 1 or type 2AX fibers in the composite fiber typing stains (Fig 4f).

### Transcriptomics

#### eMD differential gene expression.

**Fig 5:**
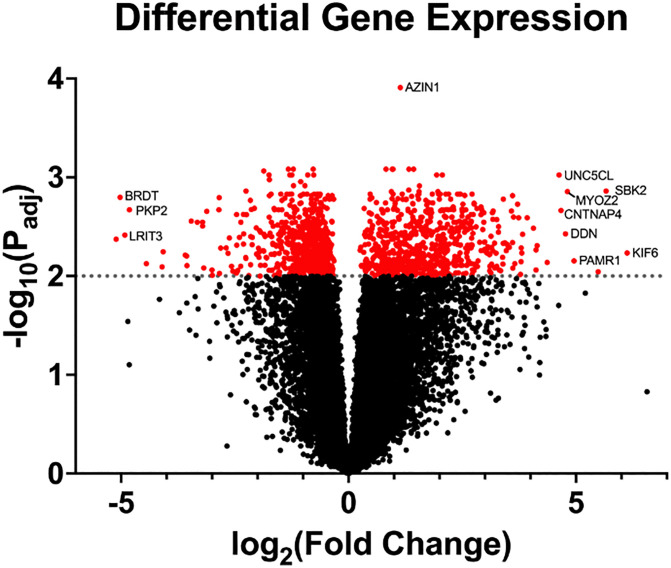
Transcriptomic differential gene expression in eMD horses. Volcano plot depicting the probability of observing the estimated change in gene expression (-log_10_ scale) on the Y axis and the degree of fold change differences (log_2_ scale) on the X axis. Selected genes are labelled.

Out of 15,697 expressed gene transcripts, there were 1442 DEG of which 155 were novel transcripts (Fig 5, S1 Table). Approximately equal numbers of DEG were up (n = 730) and down regulated (n = 712) in eMD versus controls (range: −5.71 to 6.13 log_2_ FC, FDR ≤ 0.05).

Four of the top 10 DEG with the highest or lowest log_2_FC, were involved in myogenesis including, myozenin 2 (*MYOZ2,* log_2_ FC 4.81) that modulates calcineurin signaling in type 1 fibers and thereby impacts muscle development and fiber type [15], SH3 domain binding kinase family member 2 (*SBK2* and *SBK3* log_2_ FC 5.66) involved in myocyte differentiation and sarcomere organization [16], and peptidase domain containing associated with muscle regeneration 1 (*PAMR1,* log_2_ FC 4.96) involved in muscle regeneration (Fig 5, Table 2) [17,18].

**Table 2:** Top upregulated (>3.4 Log_2_ Fold change) and down regulated (< 3.5 Log_2_ Fold change) genes in the transcriptomic analysis.

Gene Name	Encoded Protein	Function	Log_2_FC	P adjusted
UPREGULATED				
**Muscle morphogenesis**				
SBK2, SBK3	SH3 Binding Kinase 2, and 3	Regulator of muscle differentiation	5.66	1.38E-03
PAMR1	Peptidase Domain Containing Associated with Muscle Regeneration 1	Satellite cell proliferation	4.96	7.02E-03
MYOZ2	Myozenin 2	Z-disk protein regulating fiber type via calcineurin	4.81	1.40E-03
**Neurons**				
KIF6	Kinesin Family Member 6	Promotes cell proliferation in skeletal muscle development	6.13	5.84E-03
DDN	Dendrin	Postsynaptic protein involved in neuronal plasticity	4.77	3.74E-03
CNTNAP4	Contactin Associated Protein-Like 4	Synaptic adhesion protein	4.67	2.17E-03
UNC5CL	UNC5C-Like	Axonal guidance, Inhibition of NF-κB	4.63	9.46E-04
NCAM1	Neural Cell Adhesion Molecule 1	Mediates neurite outgrowth, synaptic plasticity, and cell adhesion.	4.06	3.28E-03
B3GALT1	Beta-1,3-Galactosyltransferase 1	Biosynthesis of glycolipids, myelin components	4.12	5.43E-03
KCNN1	Potassium Calcium-Activated Channel Subfamily N Member 1	Regulates neuronal excitability	3.83	3.78E-03
CPE	Carboxypeptidase E	Neurotrophic factor that promotes neuronal survival	3.79	7.15E-03
**Extra Cellular Matrix and Collagen**				
THBS2	Thrombospondin 2	Angiogenesis and collagen fibrillogenesis	4.15	4.93E-03
FNDC10	Fibronectin Type III Domain Containing 10	ECM-related protein	3.78	9.63E-03
ASPN	Asporin	ECM remodeling	3.8	5.20E-03
COL12A1	Collagen Type XII Alpha 1 Chain	Major fibrillar collagen	3.93	2.57E-03
**Miscellaneous**				
ADGRG2	Adhesion GPCR G2	Epididymis-specific transmembrane protein	4.12	8.67E-03
**DOWN REGULATED**				
**Gene**	**Name**	**Encoded protein function**	**Log2FC**	**adj.P.Val**
**Transcription, cell cycle regulation**				
BRDT	Bromodomain Testis Associated	Regulates gene expression in testis	−5.02	1.59E-03
**Neuromuscular development**				
LRIT3	Leucine-Rich Repeat, Ig-Like and Transmembrane Domain 3	May regulate fibroblast growth factor receptors (Kim)	−4.92	3.85E-03
PKP2	Plakophilin 2	Cell–cell adhesion, may play role in muscle hypertrophy (Verbrugge)	−4.82	2.13E-03
**Inflammation, protein folding**				
IL32	Interleukin 32	Pro-inflammatory cytokine	−3.56	6.28E-03
**Miscellaneous**				
OPRD1	Opioid Receptor Delta 1	GPCR mediating delta-opioid signaling	−4.1	8.06E-03

Among the other top 10 DEG, three have roles in neurons*:* kinesin family member 6 (*KIF6*, log_2_ FC 6.13), responsible for transporting protein complexes and mRNA along microtubules, dendrin (*DDN*, log_2_ FC 4.77), which plays a role in modulating the synaptic cytoskeleton structure, and contactin associated protein family member 4 (*CNTNAP4*, log_2_ FC 4.77), with functions connected to mitochondrial energy production and synaptic signaling (Table 2). [19]. The remaining top DEG included Unc-5 family C-terminal like *(UNC5CL*), which modulates inflammatory and apoptotic signaling [20] bromodomain testis associated (*BRDT*) which regulates chromatin remodeling and transcription in male germ cells [21], leucine -rich repeat, Ig-Like and transmembrane domains 3 (*LRIT3*) which regulates fibroblast growth factor receptors [22] and plakophilin 2 (*PKP2*), which participates in linking cadherins to intermediate filaments in the cytoskeleton [23] (Table 2).

Notably there was no differential expression of *CLCN1*, (log_2_FC −0.06, Padj = 0.89), *CELF1* (log_2_FC 0.388, Padj = 0.248), *MBLN1* (log_2_FC −0.71, Padj = 0.019) *MLBN2* (Log_2_FC −0.22, Padj = 0.265), *MLBN3* (log_2_FC 1.727, Padj = 0.047) or the sodium channel *SCN4A* (Log_2_FC = −0.4584; Padj = 0.1158).

### Gene ontology (GO) pathways

#### Upregulated genes.

**Fig 6:**
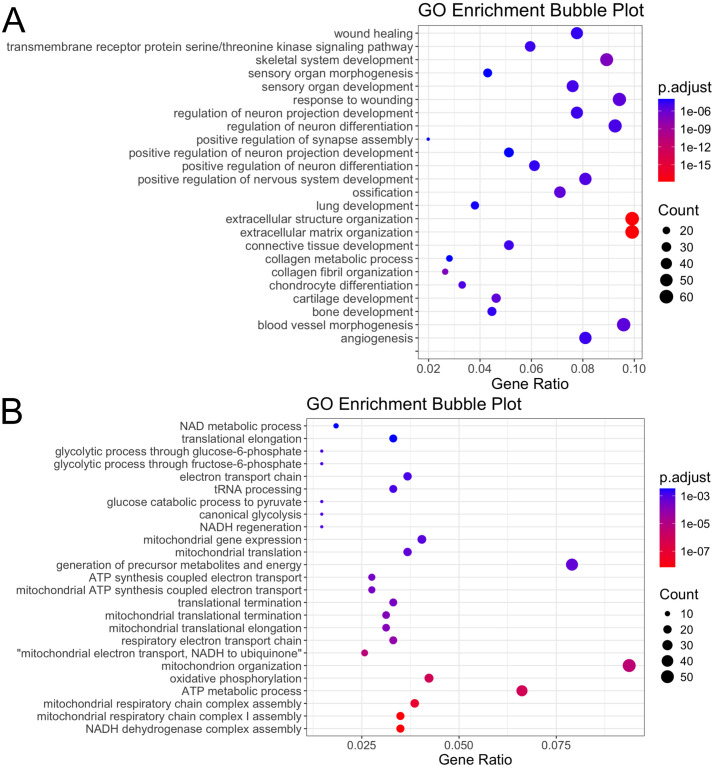
GO enrichment bubble plots. **(A)** GO enrichment bubble plots showing the GO terms for the top 25 upregulated DEG with the lowest adjusted P values on the y axis and gene ratios on the X axis. **(B)** GO enrichment bubble plots showing the GO terms for the top 25 down regulated DEG genes.

Biological Process: Of the 1450 DEG used in the enrichment analysis, there were 335 significant enriched GO terms for biological processes with 271 being upregulated relative to background expression comparing eMD versus control (S1 Table). Within biological processes the largest number of upregulated GO terms involved embryogenesis and morphogenesis/ development which, surprisingly, were not restricted to skeletal muscle but involved 15 different mesodermal-derived tissues (Fig 6a, S1 Table). The extracellular matrix and collagen along with wound healing and regulation of neuronal projections and neuronal differentiation were also prominent enriched GO terms (Fig 6a).

DEG common to wound healing and numerous morphogenesis GO terms included (1) Wnt Family Member 5A (*WNT5A*,log_2_FC 2.83, Padj 8.00E-03) which preferentially activates myogenesis through a Myf5-dependent pathway, [24] (2) Transcription factor SOX-9 (*SOX9*, log_2_FC −1.47, Padj 4.50E-03), which controls musculoskeletal system development [25] (3) Fibroblast growth factor receptor 1 (*FGFR1,* log_2_FC 1.76, Padj 1.80E-03), which regulates myoblast proliferation and differentiation [26] (4) Transforming growth factor beta receptor 2 (*TGFBR2*, log_2_FC 1.76, Padj 2.85E-03) and Transforming growth factor beta 2 (TGFB2, log_2_FC 1.5, Padj 9.39E-03), both controlling myogenic differentiation and myoblast fusion [27], and (6) Zinc finger transcription factors that function in the hedgehog signaling pathway (*GLI2*, log_2_FC 1.37, Padj 3.77E-03: *GLI3*, log_2_FC 1.75, Padj 2.11E-03) necessary for early skeletal myocyte and cardiomyocyte development (S1 Table). [28] The encoding proteins for these DEG, however, were not identified by our proteomic analysis.

*Cellular components* There were 16 enriched GO terms identified for upregulated DEG in eMD horses regarding cellular component (S1 Table). These GO terms largely involved the extracellular matrix and basement membrane, vesicles, cell substrate and junction/ focal adhesion (S1 Table).

Molecular function: There were 40 significant GO terms enriched in molecular function in upregulated DEG comparing eMD with control horses relative to background expression with functions including growth, extracellular matrix, transmembrane, receptors, cytokines, glycosaminoglycans among others (S1 Table).

#### Go terms for down regulated genes.

Down regulated GO terms (n = 69) for biological processes largely involved the mitochondria, glycolytic processes, and translation (Fig 6b). There were 22 enriched GO terms identified for down regulated DEG in eMD horses regarding cellular component which primarily involved mitochondria, ribosomes (S1 Table). There were 5 significant GO terms enriched in molecular function in down regulated DEG comparing eMD versus control horses (S1 Table) all with mitochondrial functions (S1 Table).

### Proteomics

**Fig 7:**
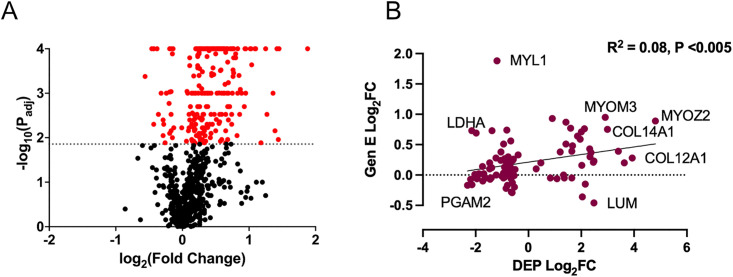
Volcano Plot for proteomic analysis and correlation to transcription. **(A)** Volcano plot depicting estimated P-values for differential protein expression versus log_2_ fold change. Significant DEP are shown in red. **(B)** Significant weak correlation between differentially expressed proteins (DEP, log_2_fold change) and expression of the encoding genes. Select proteins are labelled.

Out of 913 proteins, 27% (243) were differentially expressed (*P*_*adj*_ < 0.0139), in skeletal muscle with 89% (216) showing increased expression in eMD compared to controls (Table 3, Fig 7a and S1 Table). There was a significant weak positive correlation between DEP and gene expression (R^2^ = 0.08, P < 0.005) (Fig 7b).

**Table 3:** Gene names, the total number of differentially expressed proteins (DEP) with increased or decreased expression in their respective functional categories, DEP with >0.7 Log_2_ fold change (FC), and P values determined by a Benjamini-Hochberg analysis. Functional categories with DEP that did not meet the 0.5 Lg _2_FC threshold included purine nucleotides (N = 2 DEP), antioxidants (N = 4), smooth muscle (N = 4), red blood cells (N = 3), lipids (N = 3), and miscellaneous (N = 11) for a total of 166 DEP out of 913 total detected proteins.

Gene Name	Differentially Expressed Protein	DEP Log_2_ FC > 0.7 (eMD vs. Control)	Benjamini-Hochberg P-value < 0.014
**Myofiber differentiation**			
CALD1	caldesmon	1.39	0.007
LAMB2	Laminin subunit beta-2	1.27	0.003
MUSTN1	musculoskeletal embryonic nuclear protein 1	1.25	< 0.0001
TMSB4X	thymosin beta-4	1.04	0.00023
MYL2	Cluster of myosin regulatory light chain 2, ventricular/cardiac muscle isoform	1.1	< 0.0001
CSRP3	cysteine and glycine-rich protein 3	1.03	< 0.0001
PDLIM1	PDZ and LIM domain protein 1	0.83	< 0.0001
BIN1	Amphiphysin-2	0.76	0.00042
**Transcription and nucleosome**			
HIST1H1C	Cluster of histone H1.2	1.45	< 0.0001
MYG	MYG1 exonuclease	1.44	0.011
HIST1H1B	histone H1.5	0.85	0.0004
APOBEC-2	C-_U-editing enzyme APOBEC-2	0.77	< 0.0001
EIF4G1	eukaryotic translation initiation factor 4 gamma 1	0.77	0.00035
HMGB1	Cluster of high mobility group protein B1	0.76	< 0.0001
**Translation**			
RPL3-like	60S ribosomal protein L3-like	0.95	0.001
Rpl6	60S ribosomal protein L6	0.83	0.001
RPS20	40S ribosomal protein S20	0.8	0.00013
RPL15	60S ribosomal protein L15	0.8	0.00016
EIF4G1	eukaryotic translation initiation factor 4 gamma 1	0.77	0.00035
**Cell cycle regulation**			
S100A4	protein S100-A4	1.36	0.001
S100A1	protein S100-A1	0.93	0.002
ERH	enhancer of rudimentary homolog	0.82	0.009
**Sarcomere**			
MYL6B	Cluster of myosin light chain 6B	1.88	< 0.0001
TNNI3	troponin I, slow skeletal muscle	1.42	< 0.0001
FHL1	Cluster of four and a half LIM domains protein 1	1.09	< 0.0001
TNNC1	troponin C, slow skeletal and cardiac muscles	1.02	< 0.0001
MYOZ2	myozenin-2	0.95	< 0.0001
TNNT1	troponin T, slow skeletal muscle	0.94	< 0.0001
MYOM3	myomesin-3 Type 2 A fibers	0.87	0.00082
TTN	Cluster of titin	0.86	0.003
CRYAB	alpha-crystallin B chain	0.83	< 0.0001
PDLIM1	PDZ and LIM domain protein 1	0.83	< 0.0001
MYL3	Cluster of myosin light chain 3	0.77	< 0.0001
SMPX	small muscular protein	0.75	< 0.0001
**Extracellular matrix, basement and cell membrane**			
COL6A1	collagen alpha-1(VI) chain	0.75	< 0.0001
SGCD	delta-sarcoglycan	0.75	0.001
BGN	biglycan precursor	0.74	0.003
LGALS1	galectin-1	0.73	< 0.0001
FBN1	Cluster of fibrillin-1 i	0.71	0.002
**Neurons**			
COBL	protein cordon-bleu	0.89	0.001
TPPP3	tubulin polymerization-promoting protein family member 3	0.83	< 0.0001
DPYSL2	Cluster of dihydropyrimidinase-related protein 2	0.76	0.0003
**Intramuscular calcium regulation**			
HRC	sarcoplasmic reticulum histidine-rich calcium-binding protein	1.18	0.013
SRL	Cluster of sarcalumenin	0.77	0.00016
CALM3	calmodulin-3	0.74	0.005
**Fatty acids, Lipids**			
FABP4	fatty acid-binding protein, adipocyte	0.72	< 0.0001

Consistent with the high proportion of type 1 fibers in eMD horses, the most DEP were in mitochondria (n = 39) and the sarcomere (n = 37), with many sarcomeric DEP linked to slow-twitch fibers (Table 3). Other categories for DEP included extracellular matrix and basement/cell membrane (n = 26),nucleosome/transcription/cell cycle regulation (n = 17 DEP), translation (n = 19 DEP), myofiber differentiation (n = 10), cytoskeleton (n = 15), enzymes/glycogen metabolism (n = 19), cell stress/protein folding (n = 15) intramuscular calcium regulation (n = 14), neurons (n = 12) among others (Table 3).

#### Upregulated DEP.

Top proteins with > 1 log_2_FC were linked to skeletal muscle development (Table 3) included musculoskeletal embryonic nuclear protein 1 (MUSTN1) that regulates myoblast differentiation, thymosin beta-4 (TMSB4X) aiding in muscle cell development, and (FHL1) and myosin regulatory light chain 2 (MYL2) both critical for sarcomere assembly. Cysteine and glycine-rich protein 3 (CSRP3) considered a master regulator of muscle development was also a top upregulated DEP. [29] Caldesmon (CALD1) that acts as a developmentally regulated factor necessary for myoblast differentiation [30] was a top DEP along with laminin subunit beta 2 (LAMB2) that interacts with integrin α7β1 and α-dystroglycan to guide myoblast adhesion and migration. [31] The nucleosome and cell cycle regulation were represented by histone H1.2 (H1-2), exonuclease MYG1 (MYG) and protein S100-A4. Other proteins with > 1 log_2_FC included type 1 fiber sarcomeric proteins such as myosin light chain 6B (MYL6B) which stabilizes the myosin head region, and troponin I (TNN1) and troponin C (TNNT2, log_2_FC 1.0) critical for initiating contraction. (S1 Table).

#### Down regulated DEP.

The top DEP with> −0.30 log_2_FC were parvalbulin (PVALB, log_2_FC −0.56), a cytosolic Ca^2+^-binding protein downregulated in most muscle atrophy conditions [32], beta 2 glycoprotein (APOH, log_2_FC −0.4) that impacts lipid deposition in myoblasts [33], mitochondrial 3-hydroxyisobutyrate dehydrogenase (HIBADH, log_2_FC-0.4) that metabolizes valine, and calcium binding mitochondrial carrier protein alar 2 (SLC25A13, log_2_FC −0.32), which facilitate transport of solutes across the inner mitochondrial membrane (Table 3).

The proteomic analysis did not detect the proteins encoding *CLCN1*, *CELF1*, *MBLN1* or the sodium channel so differential expression could not be assessed.

### Trinucleotide repeats DMPK and CNBP

**Fig 8:**
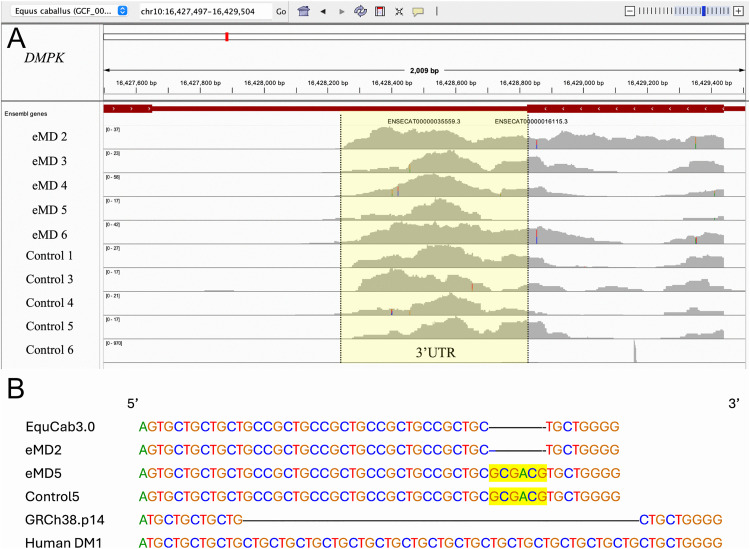
mRNA sequences in the 3’UTR of DMPK. **(A)** VCF file of mRNA sequences in the 3’ UTR of the DMPK gene in 5 eMD and 5 control horses. The yellow highlighted region represents the untranslated region for exon 1 and did not show evidence of expanded trinucleotide repeats that cause human DM1. **(B)** Alignment of the 3’ untranslated region (UTR) region of *DMPK* (EquCab 3.0) for 2 eMD and 2 control horses (RNA sequencing depth > 10 reads), compared to the same region in the human reference genome (GRch38.p14) and humans with myotonic dystrophy 1 (DM1). The highlighted region denotes some reads that had a six base pair insertion found in eMD5 and control 5.

Repeat expansion in the 3’UTR of *DMPK* (Fig 8a,b) or intron 1 of *CNBP* (S4 Fig) were not identified in our analysis of each gene.

### *CLCN1* and *SCN4A* sequence

The sequence of *CLCN1* obtained from transcriptomic data contained 7 3’UTR variants and 5 synonymous variants in coding sequences, none of which segregated with disease state (Table 4). Because SCN4A can cause myotonia [34], it was also evaluated. The *SCNA4* gene contained 12 3’ UTR variants, 6 synonymous variants, 2 missense variants and one 5’ UTR variant, none of which segregated with disease state (Table 4).

**Table 4:** The *CLCN1* and *SCN4A* variants identified in the transcriptomic data listing the chromosomal location, the reference or wild type variant, the number of horses with the variant in control and equine myotonic dystrophy (eMD) horses and the predicted consequence of the variant.

*CLCN1 mRNA* Variants					
Location	Refer-ence	Variant	Variant controls (N, %)	Variant eMD (N, %)	Predicted Consequence
			N = 5	N = 5	
4:96,504,015	C	A	1 (20%)	1 (20%)	synonymous variant
4:96,509,984	C	T	0	1 (20%)	synonymous variant
4:96,510,008	C	T	0	1 (20%)	synonymous variant
4:96,516,401	C	T	0	1 (20%)	synonymous variant
4:96,518,410	A	G	5 (100%)	4 (80%)	synonymous variant
4:96,526,804	A	G	3 (60%)	1 (20%)	3 prime UTR variant
4:96,527,208	A	G	4 (80%)	1 (20%)	3 prime UTR variant
4:96,527,350	C	T	2 (40%)	0	3 prime UTR variant
4:96,527,398	G	C	2 (40%)	1 (20%)	3 prime UTR variant
4:96,527,509	G	T	1 (20%)	1 (20%)	3 prime UTR variant
4:96,527,673	A	G	4 (80%)	1 (20%)	3 prime UTR variant
4:96,527,685	C	G	4 (80%)	1 (20%)	3 prime UTR variant
***SCN4A***					
11:15,448,572	C	T	5, 100%	3, 60%	5 prime UTR variant
11:15,452,445	C	T	2, 40%	3, 60%	Missense variant P/L
11:15,454,754	G	A	1, 20%	0, 0%	Synonymous Variant L
11:15,457,612	C	T	3, 60%	3, 60%	Synonymous Variant T
11:15,469,298	A	C	4, 80%	4, 80%	Synonymous Variant T
11:15,470,109	T	C	5, 100%	5, 100%	Splice region, synonymous variant D
11:15,475,642	T	C	3, 60%	3, 60%	Synonymous Variant T
11:15,475,756	C	T	0, 0%	1, 20%	Synonymous Variant F
11:15,476,296	C	T	3, 60%	3, 60%	Synonymous Variant P
11:15,476,298	T	C	3, 60%	3, 60%	Missense Variant L/P
11:15,476,764	G	C	0, 0%	2, 40%	3 prime UTR variant
11:15,476,908	A	G	0, 0%	2, 40%	3 prime UTR variant
11:15,477,091	G	A	2, 40%	3, 60%	3 prime UTR variant
11:15,477,103	T	G	3, 60%	3, 60%	3 prime UTR variant
11:15,477,220	G	C	5, 100%	5, 100%	3 prime UTR variant
11:15,477,290	G	A	2, 40%	0, 0%	3 prime UTR variant
11:15,477,393	T	A	5, 100%	5, 100%	3 prime UTR variant
11:15,477,445	C	T	2, 40%	0, 0%	3 prime UTR variant
11:15,477,455	G	A	1, 20%	0, 0%	3 prime UTR variant
11:15,477,458	A	T	3, 60%	3, 60%	3 prime UTR variant
11:15,477,522	A	G	5, 100%	5, 100%	3 prime UTR variant
11:15,477,801	G	A	1, 20%	1, 20%	3 prime UTR variant

### Alternative splicing

#### CLCN1.

**Fig 9:**
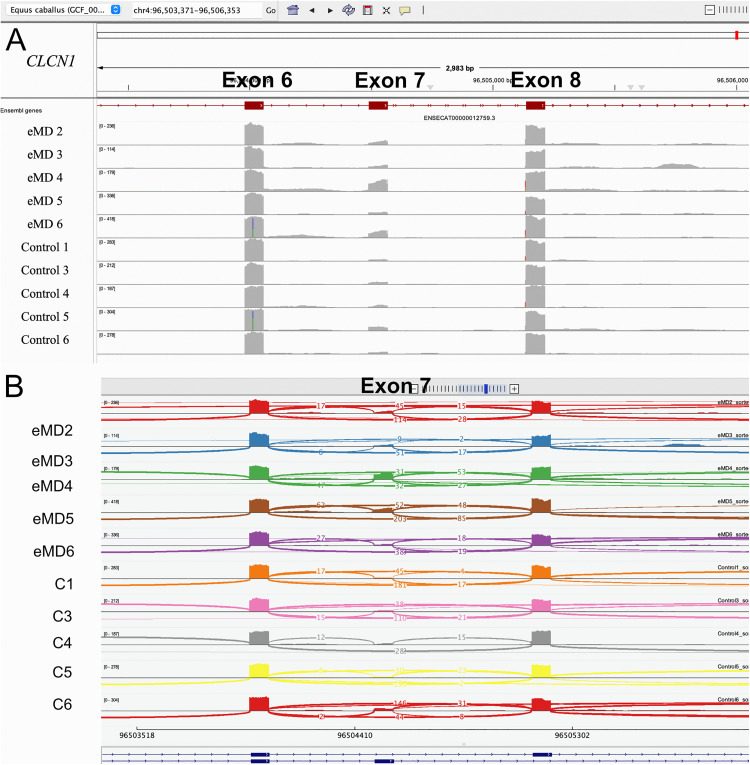
Expression of exon 7 in CLCN1. **(A)** mRNA sequences in the region of *CLCN1* exon 7 in 5 eMD and 5 control horses showing no clear evidence of retention of exon 7 exclusively in eMD versus control horses as seen in human DM1. **(B)** A sashimi plot depicting spicing analysis of exon 7. The read density is expressed as a horizontal histogram and splice junction reads are shown as arcs connecting exons with the thickness representing read counts.

**Fig 10:**
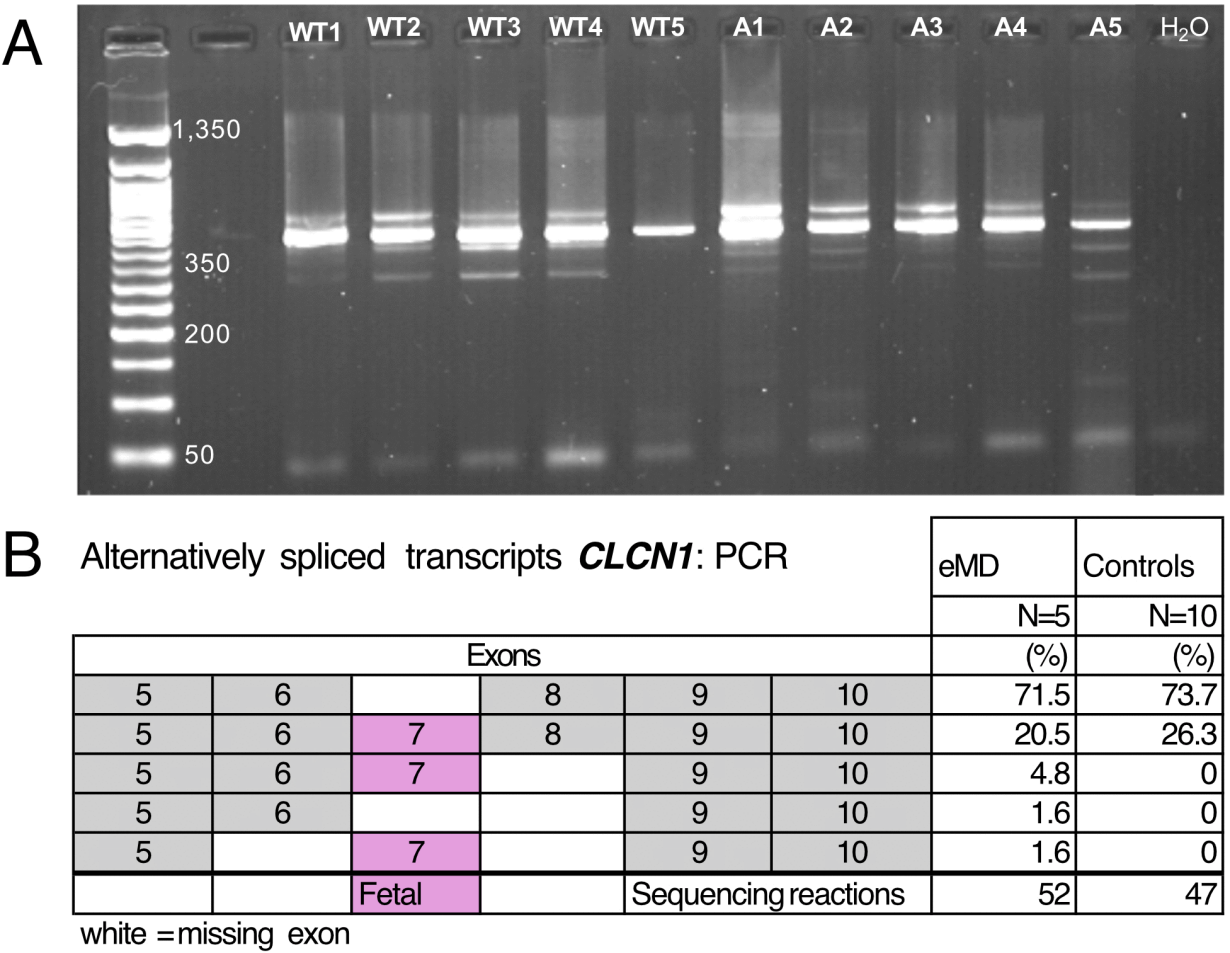
PCR amplification of CLCN1 and evaluation of alternative splicing. **(A)** Agarose gel of PCR products from amplification of *CLCN1* exons 5 through 10. WT = control horses, A1-A5 represents individual eMD horses, WT1 through 5 represent controls. **(B)** The frequency of the C*LCN1* isoforms containing variations of exons 5 through 10 in eMD and control horses. Values are expressed as the mean percentage of the total sequenced transcripts.

Examination of mRNA sequences in the VCF files and Sashimi plots for retention of exon 7 of *CLCN1* identified variable expression of exon 7 in both eMD and control horses (Fig 9a,b). RT-PCR sequencing of *CLCN1* exons 5–10 identified multiple cDNA (Fig 10a). There was a major band at 420 bp and several minor bands in all eMD and control horses (Fig 10a). Sequencing of these bands found an alternatively spliced *CLCN1* transcript in each band. In eMD and control horses, approximately 70% of *CLCN1* transcripts were functional *CLCN1* transcripts with an intact reading frame (no exon 7) and 30% of transcripts contained a premature stop codon (exon 7 included) (Fig 10b). Three additional non-functional splice variants were observed within the eMD affected horses, albeit at a very low frequency (Fig 10b). Alternative splicing of *CLCN1* did not segregate with affected status, sex, or breed. Sashimi plots covering the full *CLCN1* gene (S5 Fig) did not reveal clear evidence of missplicing; however, further detailed analysis is necessary to fully exclude this possibility.

#### ATP2A1.

Examination of mRNA sequences in the VCF files and Sashimi plots (S6 Fig) did not identify splicing of exon 22 in *ATP2A1* encoding SERCA1, which was confirmed by RT-PCR (S7 Fig) contrasting DM1 where exon 22 in SERCA1 is absent.

## Discussion

The major finding from our study was that eMD hindlimb muscles have dystrophic changes and altered fiber type composition and distribution that appear to be related to aberrant morphogenesis and regulation of myofiber development and innervation. This interpretation is substantiated by the presence of embryonic myosin (MYH3) in hindlimb muscles of adult horses, upregulation of proteins involved in the nucleosome, transcription, translation, myoblast differentiation, and axonal guidance, as well as upregulated DEG related to morphogenesis of the neuromuscular system. Unlike, human DM1 and DM2, the underlying basis for eMD does not appear to be a repeat expansion in the 3’ UTR of *DMPK* or in intron 1 of *CNBP.* Alternative splicing of *SERCA1* to exclude exon 22 and consistent retention of fetal exon 7 in *CLCN1*, characteristics observed in human DM1 were not consistent across eMD horses.

During development, primary myoblasts emerge expressing myosin heavy chain 3 (MYH3) and typically evolve into slow (MYH7) twitch fibers. [35]. Primary fibers serve as a scaffold for development of secondary fetal myoblasts expressing MYH8 that mature into type 1 (MYH7), 2A (MYH2) and, type 2X (MYH1) myofibers. [35] Unusual features of eMD muscles included the predominance of type 1 fibers and the expression of a small number of MYH3 positive fibers in hindlimb muscles of 4/6 eMD horses. In healthy foals, the percentage of gluteal myofibers expressing MYH3 quickly declines to < 1% by 2 weeks of age, contrasting their presence in 3-year-old eMD horses. [36,37]. One explanation for MYH3 expression could be the presence of regenerating fibers following myonecrosis. This seems unlikely, however, because little to no myonecrosis was evident in eMD muscle samples histologically, and there was little evidence of regeneration based on the absence of small basophilic fibers with large myonuclei and dark desmin-staining. The presence of myofibers expressing MYH3 in eMD horses at 2–3 years of age and predominance of grouped type 1 fibers more likely suggests abnormal myofiber development is a key feature of eMD. [38]

Myocyte morphogenesis and fiber type differentiation are stringently regulated by the sequential expression of transcription factors such as MyoD, Myf5, myogenin, and MRF4. [39] They govern myogenic lineage commitment, maintenance of progenitor cells, and the timing of differentiation. Numerous DEG and DEP that influence the expression of these transcription factors were identified when eMD was compared to control muscle. Firstly, eMD muscle had differential expression of proteins exerting epigenetic control of muscle development. Both DEP SMYD1 (log_2_FC 0.34) which methylates and stabilizes histone H2A.Z1 and H2A.Z1 (log_2_FC 0.38) were upregulated. H2A.Z1 expression blocks myoblast differentiation by disrupting MyoD expression. [40] Further ANP32B (log_2_FC 0.42) was upregulated which mediates the dissociation of H2A.Z1 from the nucleosome. [41,42]. H1.5 (log_2_FC 0.85) also had increased expression in eMD muscle and this histone binds to the core enhancer region of MyoD, resulting in a closed chromatin conformation, preventing MyoD activation and hindering myotube formation. [41–43] Therefore, these findings are suggestive of epigenetic down regulation of MyoD expression in eMD muscle.

Caldesmon (CALD1, log_2_FC 1.4) was a top upregulated protein that acts as a developmentally regulated factor, increasing during myoblast differentiation and necessary for myoblast differentiation. [30] Thymosin beta4 (log_2_FC 1.04) also showed increased expression in eMD muscle and it supports stem/progenitor cell mobilization, migration, and differentiation [44]. Other DEP influencing muscle fiber differentiation included upregulation of MUSTN1 (log_2_FC 1.3), which promotes the fusion of myoblasts into myotubes through MyoD and myogenin expression. [45] CSRP3 (log_2_FC 1.0), which promotes myogenesis, was also upregulated and it regulates muscle-specific gene expression through interactions with MyoD and MRF. [46] Thus, taken together there appears to be dysregulation of proteins impacting myocyte formation and differentiation in eMD compared to control gluteal and semimembranosus muscles.

Further evidence of disrupted muscle morphogenesis came from the enriched GO terms in biological processes for DEGs. There were numerous GO terms for regulation of and morphogenesis of mesodermal-derived tissues including skeletal muscle in eMD compared to control muscle. Enriched signaling pathways in eMD muscle included Wnt, Notch, and TGF-β that orchestrate specification, migration, and differentiation of mesenchymal stem cells. [47] In skeletal muscle, the precise progression of muscle precursor cells along the myogenic lineage pathway is impacted by the temporal balance between Notch and Wnt signaling, which modulates Myf5 and MyoD. [47] *NOTCH2* (log_2_FC 1.9) and *RBPJ* (log_2_FC 0.8) were both upregulated DEG. *RBPJ* is a transcription factor that binds to and activates Notch which then plays an inhibitory role in myogenic differentiation ensuring that muscle progenitors proliferate before committing to differentiation and target gene transcription. [48] *WNT5A* (log_2_FC 2.8) was also an upregulated DEG in eMD horses present in13/15 GO terms for morphogenesis of mesenchymal-derived tissues. [49] *WNT5A* promotes myogenic differentiation and muscle fiber formation. Thus, early signaling for muscle development appears to be altered in the muscle of 2 month to 3-year-old eMD horses. These enriched signaling pathways involve numerous mesenchymal derived tissues in addition to muscle, such as heart, bone and cartilage, however, the clinical signs in our eMD horses and post-mortem evaluation of previous cases suggested that morphogenesis of skeletal muscle is primarily impacted in eMD with some horses having testicular atrophy and lenticular cataracts. [3–5,7]

Other genes impacting muscle morphogenesis were also DEG in eMD versus control muscle. *miR-24* (log_2_FC 1.5) expression was significantly increased, miR-24 modulates TGF-beta-dependent inhibition of myogenesis and facilitates the transition from proliferating myoblasts to differentiated myotubes. [50] miR-24 also targets and downregulates the DEG *HMGA1* (log_2_FC −1.04) (High Mobility Group AT-Hook 1), a myogenesis inhibitor. Other upregulated DEG involved in signaling included *TGFB1* (log_2_ FC 1.7), *TGFB2* (log_2_ FC 1.5) *TGFBR2* (log_2_ FC 1.8), *TGFBR3* (log_2_ FC 1.5). TGF-β1 does not affect embryonic myoblasts but does inhibit the differentiation of fetal myoblasts by binding to its receptors and repressing MyoD and myogenin. [51] Overall, these DEG support dysregulation of genes essential for myoblast development and fusion into myotubes in eMD horses.

Type 1 fiber predominance was a feature of eMD muscle in our study and in other studies of eMD horses. [5,10,52] Myotubes formed before the expression of TGF-β1 develop into slow primary myofibers, whereas fast fibers form from secondary myoblasts particularly those adjacent to connective tissue expressing TGF-β1. [52] Together with aberrant differentiation, altered TGF-β1’s spatial and temporal expression in developing connective tissue could have contributed to the 6-fold higher percentage of type 1 fibers and fewer type 2X fibers in eMD hindlimb muscle compared to control muscle. It could also have contributed to the increase in connective tissue within muscle samples. Thus, one explanation for low percentage of type 2X fibers could be altered timing of TGF-β1 expression and its impact on potential development of type 2 muscle fibers.

In addition to type 1 fiber predominance, fiber type grouping was a prominent feature of eMD muscle in our study and other studies of eMD. [3,5,9]. This pattern is not described as a common feature of DM1 or DM2. Fiber type grouping usually arises from reinnervation following denervation, where adjacent nerve branches develop axonal sprouts that innervate nearby denervated fibers resulting in groups of fiber of the same type. [53] In all but one eMD foal in our study, there was no clinical evidence of gross muscle atrophy or prominent weakness typical of a peripheral neuropathy. Another potential source of fiber type grouping in the eMD horses could be abnormal development and distribution of motor axons. Muscle fiber type and MyHC expression remains plastic until myofibers are mono-innervated and incorporated into a motor unit. [54] In developing muscle, several gene products guide motor axons toward myotubes and, once the neuromuscular junction is established, the velocity of the innervating nerve determines fiber type and myosin heavy chain expression. Ephrin-A3 (*EFNA3*, log_2_FC 2.62), a DEG in eMD muscle, plays a crucial role in promoting and maintaining type 1 muscle fiber type during postnatal development and reinnervation. [54] Ephrin-3 does so by inhibiting the innervation of slow myofibers by fast motor axons via repulsive interactions with the EphA3 receptor. The increased expression of EFNA3 could also have arisen from the fact that it is exclusively expressed in type 1 fibers which predominated in eMD muscle. [54] MECP2 (log_2_ FC 0.62), was also a DEP in eMD muscle and it is required for proper axonal elongation of motor units and synapse formation. [55] Additionally, nestin (log_2_ FC 0.34) was a DEP and it negatively regulates postsynaptic differentiation of the neuromuscular synapse. [56] Thus, it is possible that altered axonal guidance and synaptic development could play a role in the fiber type grouping so prominent in eMD muscle.

Our GO pathway analysis of eMD shares some common features with DM1 and murine models of myotonia. These include enriched pathways of calcium signaling, mitochondrial oxidative phosphorylation, glycolysis/glycogen metabolism, ribosomal proteins, translation, MyoD targets, purine nucleotides and expression of myogenic transcription factors. [57,58] However, there were major differences in the GO analyses, including the predominance of categories of morphogenesis in eMD compared to calcium signaling and calcium homeostasis dominating human and mouse models. [57,58] Further, we did not identify repeat expansions in the same regions of *DMPK* or *CNBP* as described in DM1 or DM2. While our study did not rule out the presence of repeat expansions in other regions of the genome, a previous study using fluorescent *in situ* hybridization with repetitive nucleotide probes did not detect the presence of any CUG or CCUG repeat expansions in a case of eMD. [8] Thus, there appear to be significant differences in the cause of eMD and DM1 and DM2.

Alternative splicing is a key feature of DM1 affecting numerous proteins including SERCA1 encoded by *ATP2A1* and CLCN1. We did not find the DM1 isoform that excludes *ATP2A1* exon 22 in our eMD horses. The stop codon created by *CLCN1* exon 7 retention is a key feature of DM1 and DM2 and is considered foundational for myotonic discharges. Retention of exon 7 in *CLCN1* was not identified exclusively in eMD horses. Exon 7 retention was present in 26% of *CLCN1* sequences for both eMD and control horses by RT-PCR, with no difference in the frequency of this retention. These results suggest that retention of exon 7 does not cause myotonic discharges in eMD. The findings are consistent with research on another eMD horse, where apamine—a compound known to inhibit myotonic discharges in human DM1—did not impact myotonia. [9]

CLCN1 is the main sarcolemmal chloride channel in skeletal muscle, and it is possible that other insertions or deletions in the genome or alternative splicing at other sites in *CLCN1* impact chloride channel expression. These were not obviously present in our RNAseq analysis, however and *CLCN1* was not differentially expressed in eMD versus control horses. Analysis of *CLCN1* mRNA sequence did not find any nonsynonymous mutations in *CLCN1* similar to those found in myotonia congenita. [2,59] During the late fetal and early postnatal periods, chloride channel expression increases significantly regulated by MEF2, MyoD, and neural inputs. [60] This is crucial to dampen repetitive firing of action potentials during contraction and to prevent myotonia. [60] It is possible that the myotonic discharges observed in eMD muscle are related to abnormal morphogenesis impacting the maturation of chloride channels. Unfortunately, our proteomic analysis was unable to identify the chloride channel in any of our study horses or in previous equine proteomic studies. [61–63] Future studies using Western blots to compare CLCN1 protein expression and expanded analyses of alternative splicing are warranted to further investigate the basis for eMD.

Mutations in the sodium channel *SCN4A* are also known to cause myotonia and hyperkalemic periodic paralysis in horses. [34,64] We did not find a *SCN4A* mutation that segregated with eMD in our transcriptomic analysis and, unlike eMD, horses with hyperkalemic periodic paralysis have no discernable muscle histopathology beyond potential vacuoles in a few fibers. [64]

DM1 and DM2 are dominantly inherited, often showing longer repeat expansions and earlier onset in successive generations. [65] There have been no reports of direct transmission from dam/sire to eMD offspring although very little information exists on families of eMD horses. While breeders worry eMD could be inherited in horses, only 10 cases have been recorded among Quarter Horse-related breeds from 1995–2025, with no affected siblings reported. A small number of cases have also been described in a variety of other breeds. [6,7,10] The American Quarter Horse Association (AQHA) has registered over 7 million Quarter Horses worldwide since its founding in 1940, the American Paint Horse Association 1 million horses since 1962 and the Appaloosa Horse Club 700,000 horses. The rarity of eMD cases in these popular breeds makes it highly unlikely that eMD has a Mendelian pattern of inheritance. Instead, eMD likely represents a rare imprinting or *de novo* developmental disorder.

Our study had several limitations, eMD horses (1 month to 3 years, mean 27 months) were on average 9 months younger than controls (4 months to 5 years, mean 36 months). This was due to a lack of younger healthy controls available for study. In some breeds, muscle fiber composition changes with age and training (type 1 fibers increase slightly and type 2X fibers decrease). [37] However, in Quarter Horses, the breed we studied, little change has been noted in fiber types from birth to 1 year of age [66] so age differences were not likely to have a major impact on our results. In addition, control horses in our study underwent exercise protocols, while eMD horses did not participate in training activities. Prior research on Quarter Horse training has demonstrated a reduction in gluteal type 2X fibers from 62% to 54% and a maximum of 20% type 1 fibers; by contrast, our unexercised eMD horses had even fewer (28%) type 2X fibers and more (42%) type 1 fibers, suggesting that variations in training were unlikely to account for the observed differences in fiber type composition. [67] Furthermore, unexercised eMD horses displayed a greater abundance of mitochondrial proteins and a higher proportion of oxidative-stained fibers compared to controls, indicating that training status did not appear to influence our findings. Fiber type diameters were not measured in our study which would have been ideal; however, comparisons would have been complicated by the wide age range of eMD horses. Rather, fiber sizes were evaluated subjectively comparing relative differences among fibers within a muscle section and differences were scored with a grading system. In our study, data regarding protein function were commonly extrapolated from other species and applied to horses which could also be a limitation. Further, we did not evaluate Western blots of CLCN1 or alternate splicing in our entire RNA-seq data which is an interesting future direction for research.

In conclusion, eMD presents distinct differences from DM1 and DM2 although it shares electromyographic and histopathologic similarities. The rarity of eMD, the expression of embryonic myosin and the differential expression of genes and proteins involved in regulating myofiber and axonal morphogenesis/ differentiation suggest eMD is a multifaceted *de novo* congenital myopathy impacting skeletal muscle morphogenesis.

## Materials and methods

### Criteria for inclusion

Records of the Neuromuscular Diagnostic Laboratory (NMDL) at the University of Minnesota and Michigan State University (1996–2022) were searched to identify eMD cases which resulted in the identification of 8 horses with eMD (Table 5). The inclusion criteria for our proteomic and transcriptomic studies were: (1) histopathology consistent with eMD, (2) sufficient frozen muscle samples for new analyses and (3) EMG results consistent with eMD confirmed by a neurologist. Two additional eMD cases with clinical signs and histopathology consistent with eMD but lacking EMG were used to evaluate *CLCN1* isoforms (Table 5).

**Table 5:** Breed, age and sex of equine myotonic dystrophy (eMD) and control horses as well as the samples used for histology, fiber type composition, presence of embryonic myosin, transcriptomic and proteomic analyses.

Horses	Breed	Age (months)	Sex	Histology	Fiber Typing	Embryonic Fibers	Transcriptomics and Proteomics
eMD							
**1**	AP	2	male	SM	SM	SM	
**2**	QH	3	male	SM, Glut	SM	SM	Glut
**3**	QH	36	female	SM		SM	Glut
**4**	Paint	24	female	Tri, Glut	SM	Glut, Tri	Glut
**5**	AP	36	castrated male	Tri, Glut	Glut	Glut, Tri	Glut
**6**	AP	36	castrated male	Tri, Glut		Glut, Tri	Glut
**7**	QH	1	male	SM			
**8**	QH	2	male	SM			
**Mean Age (SD)**		**17.5 ± 17.1**		**17.5 ± 17.1**	**16.3 ± 16.6**	**22.8 ± 16.4**	**27.0 ± 16.4**
**Controls**							
**C1**	QH	60	female	SM, Glut	SM	SM	Glut
**C2**	QH	48	female	SM	SM	SM	
**C3**	QH	36	female	Glut	Glut	Glut	Glut
**C4**	QH	24	female	Glut		Glut	Glut
**C5**	QH	24	female	Glut			Glut
**C6**	QH	24	male	Glut			Glut
**C7**	QH	36	castrated male	SM			
**C8**	QH	4	castrated male	SM	SM		
**C9**	QH	12	male	SM	SM		
**Mean Age (SD)**		**29.8 ± 17.3**		**29.8 ± 17.3**	**29.6 ± 22.2.**	**42.0 ± 15.5**	**33.6 ± 15.7**

AP = Appaloosa, QH = Quarter Horse, SM = semimembranosus, GT = middle gluteal, Tri = triceps brachii.

**Fig 11:**
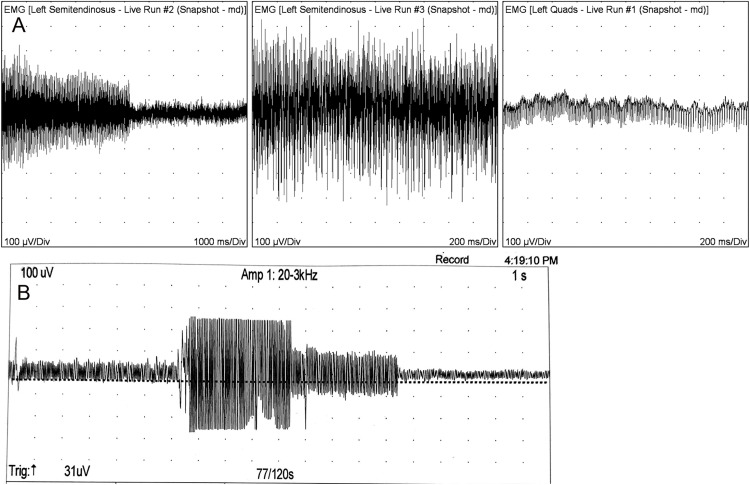
Myotonic discharges. **(A)** Diffuse waxing and waning myotonic potentials that never ceased found throughout out all muscles except the semimembranosus muscle in Horse 2. **(B)** Myotonic discharges in Horse 3.

The EMG criteria for the 6 resulting eMD horses 1–6 included myotonic potentials (high amplitude (up to 1mV), long duration (>500ms) discharges, audible as dive bombers, that ceased abruptly before initiating a new spontaneous discharge in EMG tracings (Fig 11a,b, S1 Video).

### Horses

**Fig 12:**
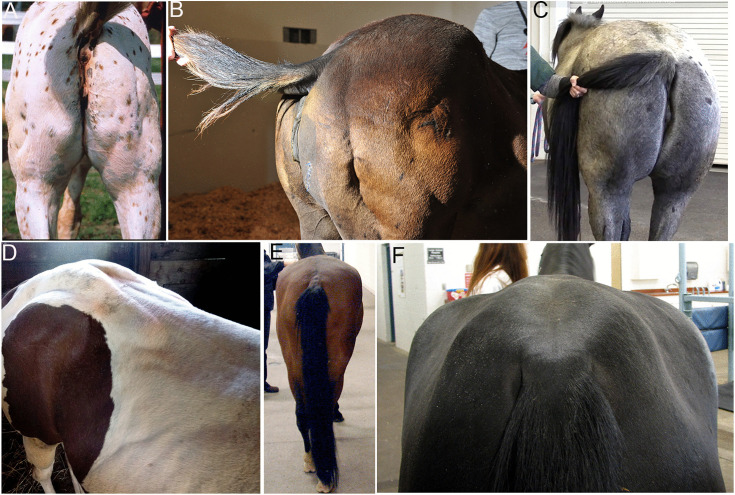
Hindquarter muscle development in eMD Horses. **(A)** Horse 1 with pronounced muscle development and focal muscle contractures in the semimembranosus muscle at 2 months of age. **(B)** Horse 2 with prominent development of the middle gluteal and semitendinosus muscles at 3 months of age. **(C)** Horse 3 with large semitendinosus and semimembranosus muscles at 3 years of age. **(D)** Horse 4 with contractures in the middle gluteal muscles at 2 years of age. **(E)** Horse 5 with progressive hypotrophy of the superficial gluteal and biceps femoris muscles at 2 years of age. **(F)** Horse 6 with progressive atrophy of the middle gluteal muscle and hypertrophy of the superficial gluteal muscle.

**eMD Horse 1** was a 2-month-old male Appaloosa that presented to the University of Minnesota for muscle stiffness and pronounced hindquarter muscle mass (Fig 12a). Progressive severe stiffness made rising from recumbency difficult resulting in the owner electing euthanasia. Neurologic examination was normal apart from prolonged firm contractures that developed after percussion of the semimembranosus and semitendinosus muscles.

**eMD Horse 2** was a 4-month-old male Quarter Horse that presented to Washington State University Veterinary Hospital with well-developed hind limb, epaxial and triceps muscles and a stiff gait (Fig 12b). The owner elected to euthanize the foal after EMG and muscle histopathology were performed due to the poor prognosis.

**eMD Horse 3** was a female Quarter Horse that initially presented to Colorado State University at one-day-of-age with lethargy, diarrhea, stiff, firm musculature in the hindquarters, elevated serum CK (7206 U/L, reference range 100–470 U/L) and AST (11,712 U/L, reference range 185–375 U/L), and deficiencies of vitamin E (serum <0.65 microg/ml; reference range 1–3 microg/ml) and selenium (0.02 ppm; reference range 0.14–0.25 ppm). The foal responded well to antibiotics, intravenous fluids, intramuscular selenium, and vitamin E injections. Genetic testing for type 1 polysaccharide storage myopathy (PSSM1) and HYPP were negative.

As a yearling, episodes of diffuse muscle spasms and mild discomfort were apparent that were managed on farm with flunixin meglumine and detomidine. By three years of age, the horse was well-muscled with prominent gluteal, semimembranosus and semitendinosus muscles and was hospitalized for a prolonged (8 hour) episode of firm muscle spasms along the neck, topline, and hindquarters with intermittent sweating (Fig 12c). Elevated serum CK (24,758 IU/L), AST (1,214 IU/L), and peripheral blood lactate (4.4 mmol/L; reference range 0.5–1 mmol/L) were noted during spasms as well as low serum vitamin E (0.7 microg/ml; reference range 2–3 microg/ml). Treatment consisted of IV fluids, flunixin meglumine (500 mg/kg IV q12h), an oral muscle relaxant (methocarbamol, 50 mg/kg PO q12h), and oral vitamin E (5,000 IU PO q24h). This resulted in a reduction in CK 3,547 IU/L over two days; however, muscle spasms persist not associated with consistent stimuli such as physical activity, weather changes, or dietary adjustments. Muscle biopsy for histopathology and EMG were performed in the hospital at 3 years of age, a subsequent percutaneous needle biopsy was obtained on the farm, and the horse now lives on pasture. Occasionally muscle spasms are seen that do not appear to interfere with his quality of life.

**eMD Horse 4** was a 2-year-old Paint mare that was donated to Michigan State University because of small stature, stiff gait, and intermittent marked spasms of epaxial and hindquarter muscles that were apparent at a few months of age (Fig 12d). The horse was euthanized after the histopathologic and EMG diagnosis of eMD.

**eMD Horses 5 and 6** were unrelated Appaloosa colts from separate farms donated to the University of Minnesota at 6 months of age because of persistent muscle stiffness. They were followed for 1.5 years prior to euthanasia. Both horses had intermittent marked unilateral hindlimb muscle contractions and stiffness at a young age that were exacerbated with exercise. Horse 5 had progressive hypotrophy of the superficial gluteal and biceps femoris muscles at 2 years of age (Fig 12e). Horse 6 had progressive atrophy of the middle gluteal muscle and hypertrophy of the superficial gluteal muscle (Fig 12f).

**eMD Horses 7 and 8** were 1- and 2-month-old Quarter Horse males presenting to the University of Minnesota from separate farms with muscle stiffness and pronounced hindquarter muscle mass. Progressive severe stiffness made rising from recumbency difficult resulting in the owner’s electing euthanasia. These two horses were used for *CLCN1* isoform analysis, Horses A1 and A2 in Fig 10.

Pedigrees were only available for eMD Horses 2 and 3 and a relationship between the two horses was not apparent within 6 generations. The sire of Horse 2 had produced 42 foals, and the sire of Horse 3 had produced 48 foals. The owner of Horse 6 said his dam had multiple previous foals that were all healthy but did not provide pedigrees.

**Control Horses** Control horses for the histologic, transcriptomic and proteomic studies consisted of 6 healthy Quarter Horses from a research herd at the University of Minnesota enrolled in an exercise study (5 females, 1 male, ranging in age from 2 to 5 years) (Table 5). Muscle submitted to the NMDL from 2 young Quarter Horses (4–12 months) and from an aborted fetus was used for muscle fiber typing of additional age matched controls. For the *CLCN1* isoform study, semimembranosus muscle from 10 additional control horses ages 2–10 years-of-age were utilized.

This study was carried out in strict accordance with the recommendations in the Guide for the Care and Use of Laboratory Animals of the National Institutes of Health. The protocol was approved by the Animal Use and Care Committee of the University of Minnesota and Michigan State University (Proto201900038). All horses were euthanized in a calm setting using rapid intravenous injection of pentobarbital sodium via a jugular catheter.

### Muscle collection

Gluteal, semimembranosus and triceps muscle samples were collected from 7 of the eMD horses immediately after euthanasia (Table 5). For the one surviving eMD horse, an open semimembranosus biopsy was used for histology, and a snap frozen percutaneous gluteal needle biopsy was used for transcriptomic and proteomic analysis (Table 5). Muscle samples from control horses were collected using a 6 mm diameter percutaneous needle biopsy or open surgical biopsy (Table 5). [68] Samples for histology were placed in saline dampened gauze and transported chilled to the NMDL where they were frozen in isopentane chilled in liquid nitrogen. Samples for proteomic and transcriptomic analyses were immediately frozen in liquid nitrogen and transported to the laboratory on dry ice. Samples were stored at −80°C until analysis.

### Muscle histopathology

Muscle samples for histopathology were sectioned 5μm thick on a cryostat and stained with hematoxylin eosin (HE), modified Gomori Trichrome, periodic acids Schiff’s (PAS), amylase PAS, oil red O, nicotinamide adenine dinucleotide tetrazolium reductase (NADH) and immunohistochemically stained for desmin. [68] Fiber size variation was evaluated relative to other fibers within the sample. Fiber splitting, fibrosis, adipocytes, sarcoplasmic masses, internalized myonuclei, acute necrosis, macrophages, regeneration, ringbinden fibers, abnormal polysaccharide, and oxidative fiber type grouping were also evaluated. Each category was scored as 0 = not present, 1 = mild alterations were present in<20% of 10x field, 2 = moderate alterations present in 21–50% of 10X field and 3 = severe alterations present in > 50% of 10X fields. Statistical comparisons were performed using a Mann Whitney test.

### Muscle fiber typing

Samples with the least freeze artifacts were selected for muscle fiber typing. Fiber types were determined by immunofluorescence on semimembranosus muscle (n = 3) and gluteal muscle (n = 1) from eMD horses and for controls, semimembranosus (n = 4) and gluteal (n = 1) muscle (S1 Table). Percentages were determined by typing at least 150 myofibers in the imunoflourescent stains. Type 1, 2A, and 2X muscle fiber types were identified by multiple fluorescent labeling according to Tulloch et al 2011. [69] Briefly, sections were incubated with a goat polyclonal anti-collagen V IgG antibody (1350-01 Southern Biotech) 1:100 for 1 hour at room temperature. Next, three separate mouse monoclonal antibodies to detect type 1, slow myosin IgG 1:100 (MAB1628 Millipore), type 2a IgG 1:6 (A4.74 DSHB) and both type 2a and 2x IgG 1:10 (NCL-MHCf Leica Biosystems) were conjugated to fluorescent IgG_1_ Fab fragments using Zenon ® Mouse IgG labeling kits (Life Technologies) Alexa Fluor® 488 (A4.74), Alexa Fluor® 594 (NCL-MHCF) and Pacific Blue™ (MAB1628). The three Zenon® labeled antibodies were admixed, added to the tissue sections and incubated at 4°C overnight. A secondary antibody for Collagen V, FITC-rabbit anti-goat IgG (61–1611, Invitrogen) 1:500 was applied to the cryosections and incubated for 1 hour at room temperature. Sections were subsequently mounted using VECTASHIELD mounting medium (H1000, Vector Labs) and examined using a fluorescence microscope (Olympus) with filters designed for each of the different emitting wavelengths. Images were captured and pseudo-colored composites generated. Fiber type composition was compared between samples using an unpaired t test.

### Embryonic myosin

Fiber typing for embryonic myosin heavy chain (MYH3) was performed on muscle samples from one aborted equine fetus (positive control), 4 eMD horses (semimembranosus n = 2, gluteal n = 2, triceps = 3) and 3 controls (2 semimembranosus, 1 gluteal) (S1, S3 Figs). Sections 10 μm thick were thawed for one hour at room temperature in slide box enclosed in foil. Cryosections were then placed in tris-buffered saline (TBS) for 15 minutes followed by three washes in tris-buffered saline with Tween-20 (TBST). Cryosections were blocked using 5% bovine serum albumin (BSA) in TBST for two hours at room temperature. Anti-MyH3, 1:50 (NCL-MHCd Leica Biosystems), was placed on cryosections and incubated at 4°C overnight. Secondary antibody (517177 Santa Cruz Biotechnology) 1:100 was applied to the cryosections and incubated for two hours at room temperature. Sections were subsequently mounted using Vectasheild Plus (H-2000 Vector Laboratories) and examined using a fluorescence microscope (Zeiss) at appropriate wavelengths. Images were captured and pseudo-colored composites generated.

### Transcriptomics

#### RNA isolation.

Total RNA was isolated from flash frozen triceps brachii, gluteus medius, and semimembranosus samples as previously described. [63] Quantification and quality of RNA was assessed using a Qubit Fluorometer and RNA HS Assay Kit (Thermo Fisher Scientific, Waltham, MA) and RNA integrity (RIN) was determined using an Agilent 2100 Bioanalyzer and an Agilent RNA 6000 Pico Kit (Agilent Technologies, Santa Clara, CA). Samples with RIN > 7.0 were used for further analysis.

#### RNA Library.

Library construction was performed with a strand-specific polyA capture protocol (TruSeq Stranded mRNA Library, Illumina, San Diego, CA) and sequencing was performed in a 2x150bp paired end format using HiSeq 4000 SBS reagents for a target of 35–40 million reads for each sample. Base calling was done by Illumina Real Time Analysis (RTA) v2.7.7 and output of RTA was sorted and converted to FastQ format with Illumina Bcl2fastq v2.19.1 for analysis.

#### Assembly and mapping.

Paired end RNA-seq reads were assessed for quality. Quality reports for raw fastq sequence files were generated using FastQC [70]. MultiQC was used to concatenate FASTQC quality reports into a single file (Ewels et al. 2016). Adapter sequences were trimmed using Trimmomatic software [71], and low quality reads (Q ≤ 30) were filtered. A splice junction mapper was used to align reads to Equcab 3.0 (National Center of Biotechnology Information https://www.ncbi.nlm.nih.gov/assembly/GCF_002863925.1/) following the stranded protocol with HISAT2 [72] The transcriptome of each sample was assembled using StringTie. [73] HTSeq was used to quantify gene expression counts. [74] Sequence data have been deposited in the NCBI Sequence Read Archive with BioProject ID PRJNA1300344.

*Expressed gene transcripts* On average 51.5 million short-read pairs (range 33.9–63.2 million) were sequenced per sample library. Adapter and quality filtering removed 17.1% of reads. The retained sequence reads were mapped to the EquCab 3.0 reference genome. Only the uniquely mapped reads were used to quantify transcript abundance (99.15% of total sequenced read pairs). The average depth of coverage per sequenced base was 36.3 with an average of 2.5 coverage depth of the reference genome. A total of 30,567 gene transcripts were expressed. After filtering for low count transcripts, 15,697 remained for the differential expression analysis.

#### Differential expression and statistics.

Differential expression analysis was conducted using a linear mixed model accounting for sex, age, muscle and diagnosis using a differential expression for repeated measures (DREAM) analysis [75] through limma/voom. [76] Genes were retained for differential expression analysis if they were present at greater than 2 reads in at least 80% of the horses. Significance was set at *P*_*adj*_ < 0.01.

#### Pathway Enrichment.

Differentially expressed genes were analyzed for functional enrichment using Gene Ontology (GO), Kyoto Encyclopedia of Genes and Genomes (KEGG), and Reactome pathway databases in R (v4.0.2). Gene identifiers were cross referenced and curated to resolve ambiguous transcript annotations and then mapped to Entrez IDs using *org.Hs.e.g.,db* (v3.11.4). [77] Enrichment analyses were conducted separately for all significant genes, up-regulated genes, and down-regulated genes. GO terms and KEGG pathways were assessed with *clusterProfiler* (v3.99.1), while Reactome enrichment was performed using *ReactomePA* (v1.32.0) with FDR-adjusted *q* < 0.05 as the significance threshold. [78,79].

### Proteomics

Protein isolation was performed on frozen muscle tissue from eMD horses (4 gluteal and 1 semimembranosus) and 6 control gluteal samples utilizing radioimmunoprecipitation assay lysis buffer (Thermo Scientific, Waltham, MA) with protease inhibitor (Roche Complete, Mini, EDTA-free, Thomas Scientific, Swedesboro NJ). Protein concentration was measured by standard bicinchoninic acid assay (Pierce^TM^ Biotechnology, Rockford, IL) and Coomassie-stained sodium dodecyl-sulfate gel. In brief, 120 µg of protein of each sample was subjected to proteolytic digestion using Trypsin/LysC enzyme mix (Promega, Madison, WI) at 1:100 (enzyme:protein) by volume. After enzymatic digestion, the samples were incubated with agitation. The samples then were acidified (2% trifluoroacetic acid), purified with c18 SepPaks (Waters, www.waters.com) and dried by vacuum centrifugation.

One hundred µg of each sample was resuspended in 100 µL of 100mM triethylamonium bicarbonate. The peptides then were tagged with TMT11 reagents (Thermo Scientific, Waltham, MA) per manufacturer’s protocol. Labelled peptides were mixed in equal portions and reverse phase C18 stagetips [80] were used to de-salt the combined sample.

One control sample was run in duplicate as an internal assay control. Tagged peptides were resuspended, washed, and eluted with the Thermo Acclaim PepMap RSLC 0.1 mm 20 mm C18 trapping column (Thermo Scientific, Waltham, MA) over 125 minutes at a constant flow rate (300 nl/min). The resulting eluted peptides were sprayed into a ThermoScientific Q-Exactive HF-X mass spectrometer (Thermo Scientific, Waltham, MA) using a FlexSpray spray ion source. The top 15 ions in each survey scan (Orbi trap 120,000 resolution at m/z 200) were subjected to higher energy collision induced dissociation with fragment spectra acquired at 45,000 resolution. The resulting MS/MS spectra were processed using Proteome Discoverer v2.2 (Thermo Scientific, Waltham, MA) to generate peak lists. Peak lists were searched against the EquCab3.0 UniProt:UP000002281 protein database appended with common laboratory contaminant (cRAP project) using Mascot v2.6 (Matrix Science, London, UK; version Mascot in Proteome Discoverer 2.2.0.388). The output then was analyzed using Scaffold (v5.0.1, www.proteomesoftware.com) to probabilistically validate protein identifications with 1% false discovery rate confidence considered true. Mass spectrometry proteomic data are available at the ProteomeXchange Consortium PRIDE repository with identifier ID:PXD066831.

Quantitative data analysis: Scaffold Q+ (v5.0.1; Proteome Software Inc., Portland, OR) was used to quantitate TMT-11 plex-labelled peptide and to probabilistically validate protein identifications. Peptide identifications were accepted if they could be established at >10.0% probability to achieve a false discovery rate (FDR) < 0.1%. Probabilities generated by Mascot were assigned by the Scaffold Local FDR algorithm. Protein identifications were accepted if they could be established at ≥ 99% probability, as assigned by the Prophet algorithm [81], and contained at least 2 identified peptides. Proteins that contained similar peptides and could not be differentiated based on MS/MS analysis alone were grouped to satisfy the principles of parsimony. Proteins sharing significant peptide evidence were grouped into clusters. Channels underwent matrix correction as reported by i-Tracker. [82]

Normalization was performed iteratively (both across samples and spectra) on intensities as described in Statistical Analysis of Relative Labelled Mass Spectrometry Data from Complex Samples Using ANOVA. [83] Medians were used for averaging. Spectra data were log-transformed and pruned of those matched to multiple proteins and weighted by an adaptive intensity weighting algorithm. Of 41336 spectra in the experiment at the given thresholds, 31211 (76%) were included in quantitation. Differentially expressed proteins were determined by applying a permutation test with unadjusted significance level of p < 0.05 corrected by Benjamini-Hochberg *P*_*adj*_ < 0.0139.

### Repeat expansions

Repeat expansions in the 3’ UTR of *DMPK* were explored by examining the sequence in region chr10: 16,427,497–16,429,504 and in VCF files. For repeat expansion in the first intron of *CNBP* the region chr 16: 2,980,584–2,990,279 was evaluated.

### Alternative splicing

Sequence: The coding sequence and partial 3’ UTR sequence of *CLCN1* and *SCN4A* was determined from transcriptomic data for 5 eMD and 5 control horses.

The region of *CLCN1* chr4:96,503,371–96,506, 353 containing exon 7 (fetal isoform) in the transcriptomic data was evaluated in a variant call format file (VCF) to determine if exon 7 was expressed in eMD versus control horses. The region of *ATP2A1* chr13:21,008,494–21,009,060 containing exon 22 to determine if exon 22 was expressed in eMD versus control horses.

Isoform expression: cDNA sequence from *CLCN1* exons 1–10, and *SERCA1* to include exon 21–23 were obtained by RT-PCR. In brief, mRNA was isolated from skeletal muscle tissue of both eMD and control Quarter Horse using Qiagen RNAeasy total RNA isolation kit (Qiagen, Valencia, CA). cDNA was prepared using the Invitrogen Superscript II RT kit with random hexamers as primer. Primers (IDT, Coralville, IA) were used to PCR amplify exons 1–10 (Table 6) PCR products were resolved by agarose gel electrophoresis, purified with Qiagen Gel Extraction kit (Qiagen, Valencia, CA) and sequenced on Applied Biosystems 3130 xl automated DNA sequencer. DNA sequences were manually edited with Sequencher software (Gene Codes Corporation, Ann Arbor, MI).

**Table 6:** PCR primers used to amplify exon 5 - 10 of *CLCN1* and exons21 −23 of *ATP2A1.*

Primer	Sequence
CLCN1ex5F	CAATTCTTCGTGGGGTTGTC
CLCN1ex9R	CCATTCGGAAATTGGTTCTG
ATP2A1EX21F	TGGTTACCATCGAGATGTGC
ATP2A123R	TCATCGAGCAAAATGACTGG

## Supporting information

S1 Fig Images of immunofluorescent staining for fiber type (type 1 blue, type 2A green, type 2X reddish brown) for 4 eMD and 5 control horses. Note the grouping of type 1 fibers in eMD horses.
(TIFF)

S2 Fig Composition of type 1, type 2A, type 2X and type 2AX fibers in gluteal and semimembranosus muscles of all horses with myotonic dystrophy (eMD) and Control horses. Means and P values are shown for significant differences between type 1 fibers in eMD versus controls and type 2X fiber types.
(TIFF)

S3 Fig Images of immunofluorescent staining for MYH3 embryonic myosin (red) in 6 eMD and 3 control horses and fetal muscle as a positive control. (TIFF)

S4 Fig mRNA sequences in the region of intron 1 of *CNBP* gene in 5 eMD and 5 control horses showing no evidence of retention of trinucleotide repeats as seen in DM2. (TIFF)

S5 Fig Sashimi from transcriptomic data depicting spicing analysis plot of entire *CLCN1* gene. The read density is expressed as a horizontal histogram and splice junction reads are shown as arcs connecting exons with the thickness representing read counts.
(TIFF)

S6 Fig Sashimi from transcriptomic data depicting spicing analysis plot of exons 21–23 of ATP2A1 encoding SERCA1. The read density is expressed as a horizontal histogram and splice junction reads are shown as arcs connecting exons with the thickness representing read counts. Unlike DM1 exon 22 was not misspliced.
(TIFF)

S7 Fig Agarose gel of PCR products from amplification of *ATP2A1 encoding SERCA1* exons 21–23. WT = control horses, A1-A5 represents individual eMD horses, WT1–5 represent controls. No alternative splicing was observed where exon 22 was excluded.
(TIFF)

S1 Table Material is provided in several tabs. Scores for muscle histopathology in gluteal or semimembranosus muscle of 6 horses with myotonic dystrophy (eMD) and controls as well as scores for triceps muscle in 4 eMD horses. Scoring system was 0 = not present, 1 = mild alterations were present in<20% of 10x field, 2 = moderate alterations present in 21–50% of 10X field and 3 = severe alterations present in > 50% of 10X fields. Statistical comparisons were performed with a Mann Whitney test. Differentially Expressed Genes in eMD versus controls (FDR ≤ 0.01) with Log_2_ fold change and adj P values. Gene ontogeny enrichment analysis for upregulated genes organized by functional categories. Gene ontogeny enrichment analysis for down regulated genes organized by functional categories. Significant differentially expressed proteins in eMD versus controls organized by functional group.
(XLSX)

S1 Video Electromyography performed on eMD horse 6 showing classic waxing and waning repetitive discharges. (MOV)
