# Supplementary figures and images for: Aberrant skeletal muscle morphogenesis and myofiber differentiation characterize equine myotonic dystrophy
Source: PLoS One. 2026 Jan 29;21(1):e0341655. doi: 10.1371/journal.pone.0341655 (PMC12854428; doi:10.1371/journal.pone.0341655)

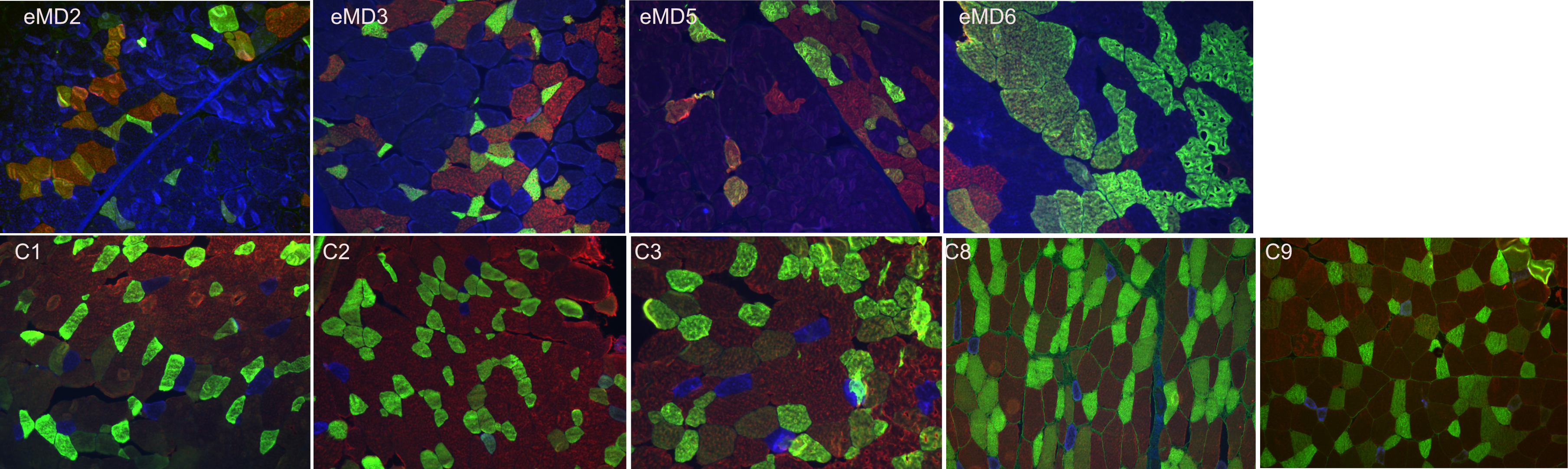

Supplement: S1 Fig — Note the grouping of type 1 fibers in eMD horses. (TIFF) [file pone.0341655.s001.tiff]

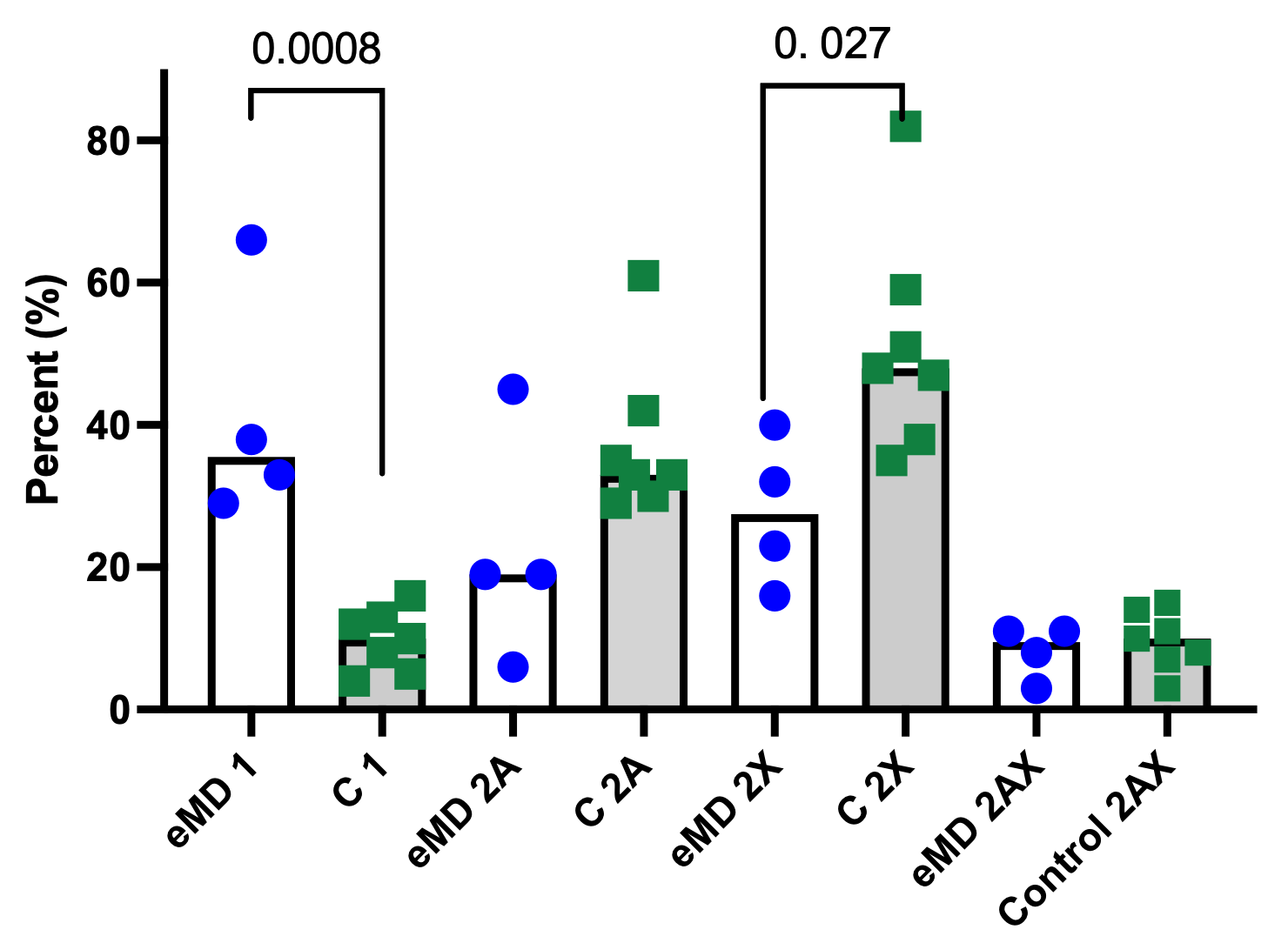

Supplement: S2 Fig — Means and P values are shown for significant differences between type 1 fibers in eMD versus controls and type 2X fiber types. (TIFF) [file pone.0341655.s002.tiff]

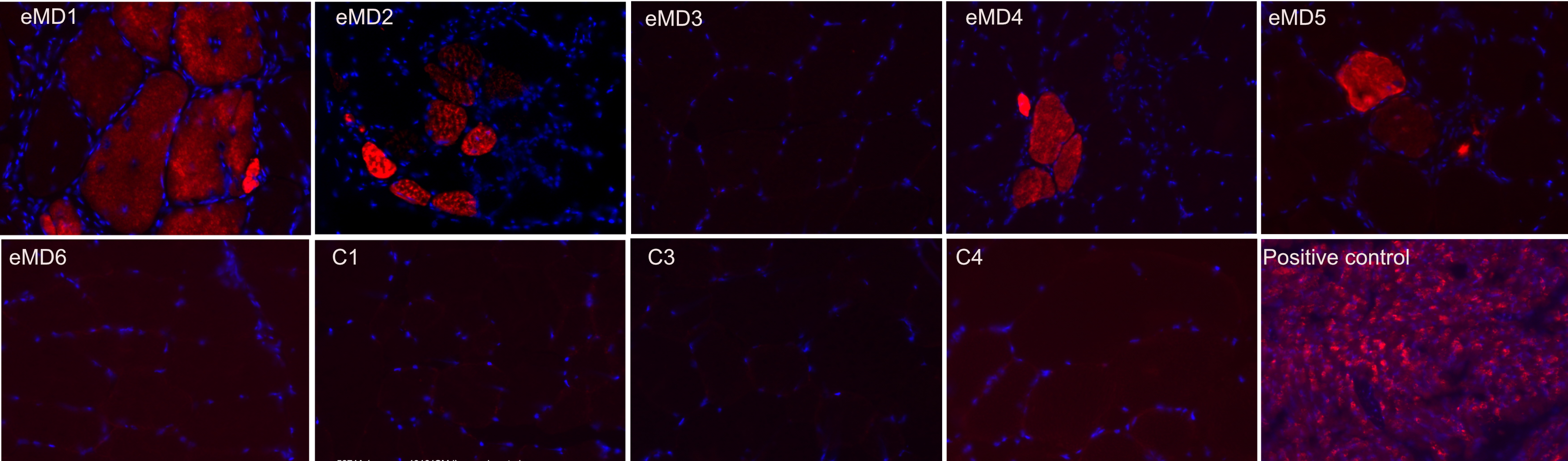

Supplement: S3 Fig — (TIFF) [file pone.0341655.s003.tiff]

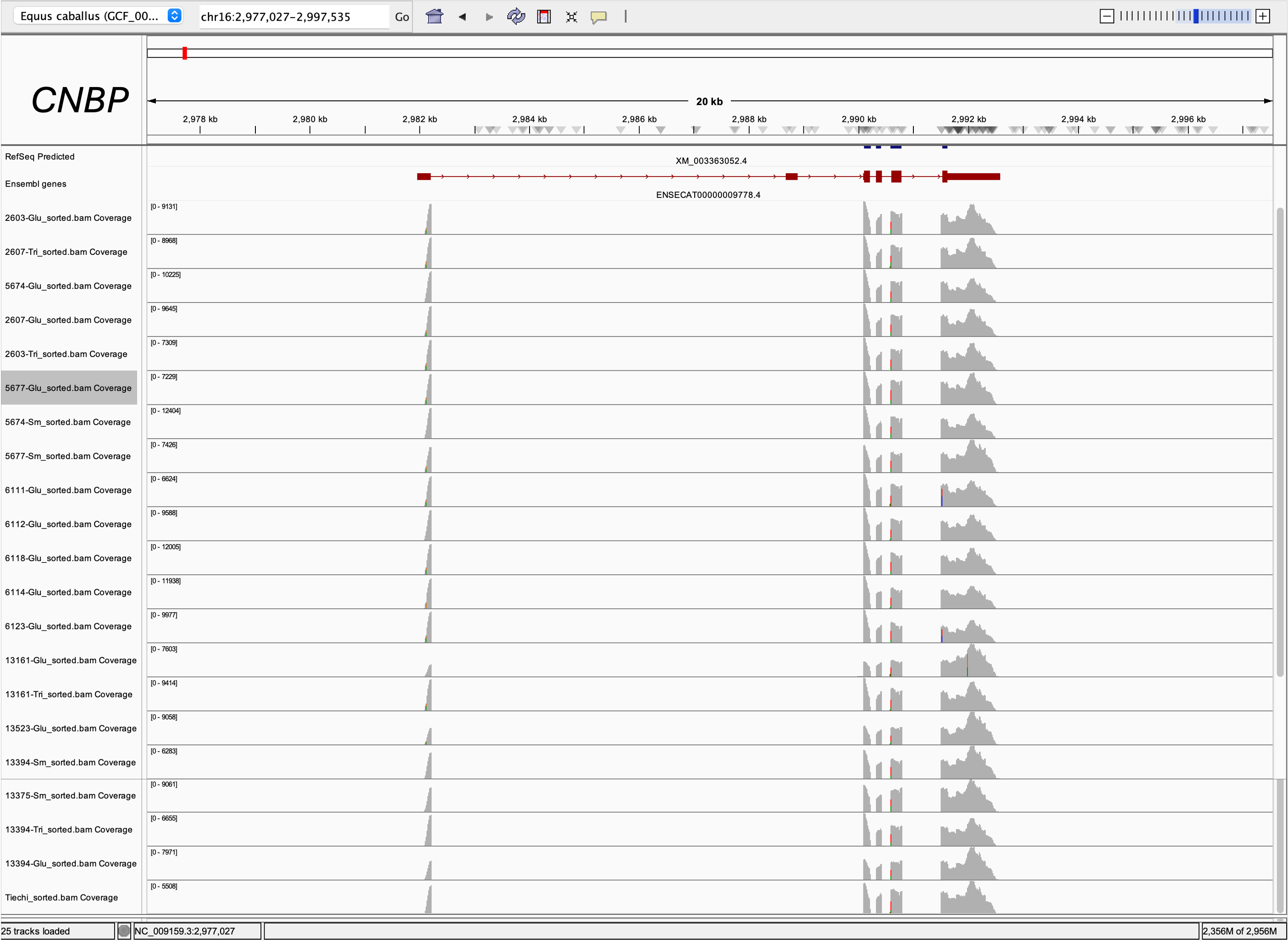

Supplement: S4 Fig — (TIFF) [file pone.0341655.s004.tiff]

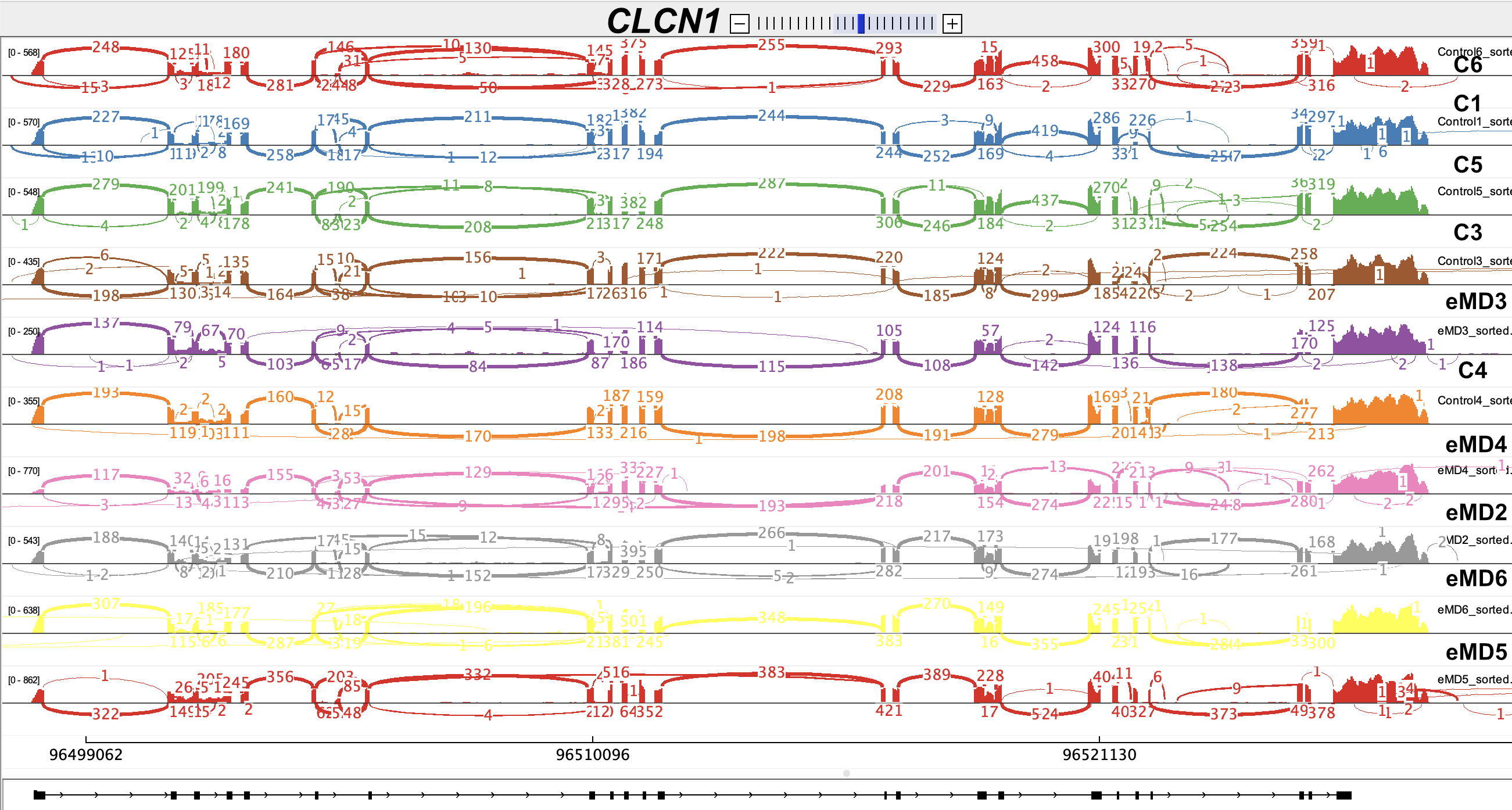

Supplement: S5 Fig — The read density is expressed as a horizontal histogram and splice junction reads are shown as arcs connecting exons with the thickness representing read counts. (TIFF) [file pone.0341655.s005.tiff]

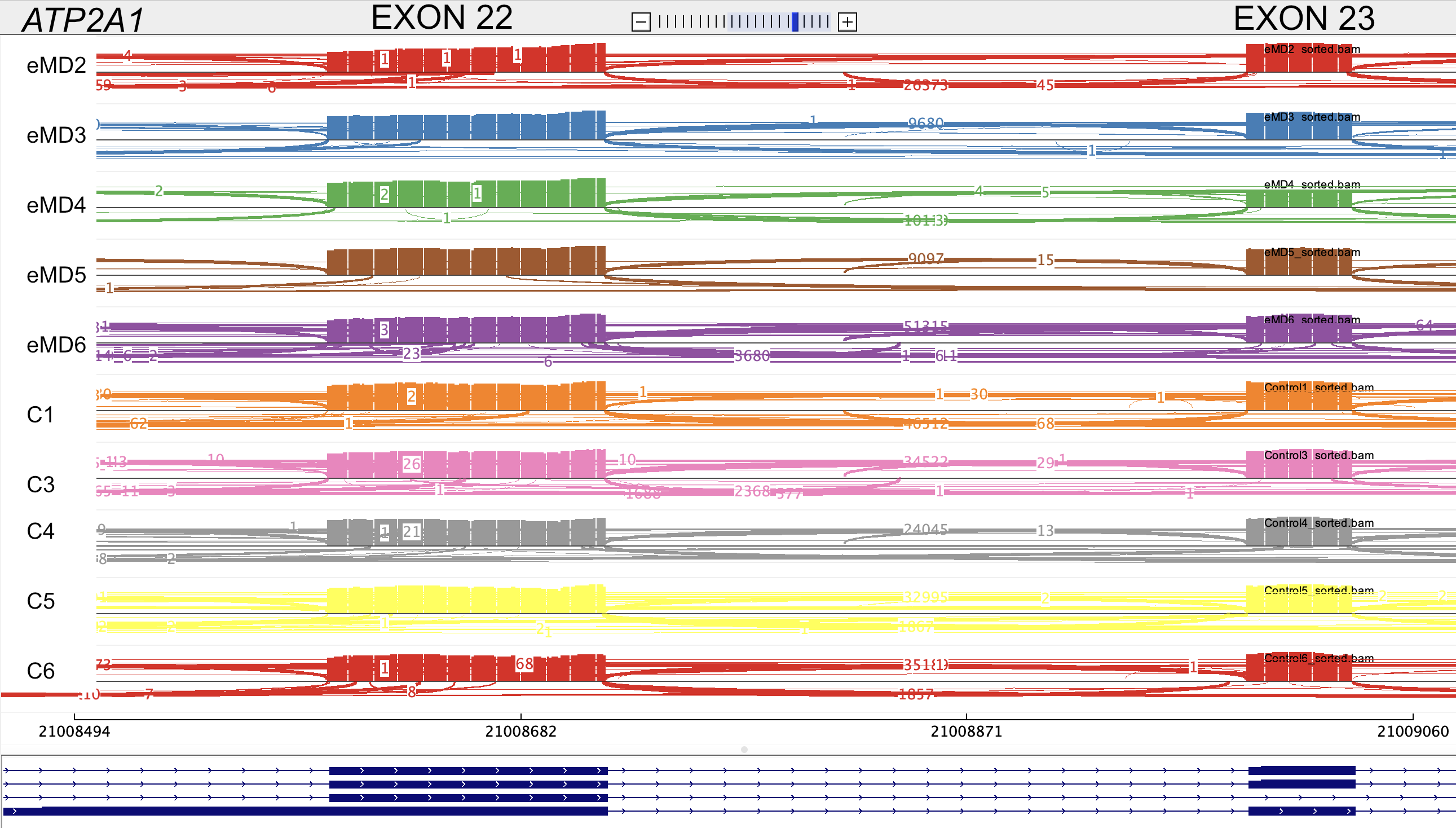

Supplement: S6 Fig — The read density is expressed as a horizontal histogram and splice junction reads are shown as arcs connecting exons with the thickness representing read counts. Unlike DM1 exon 22 was not misspliced. (TIFF) [file pone.0341655.s006.tiff]

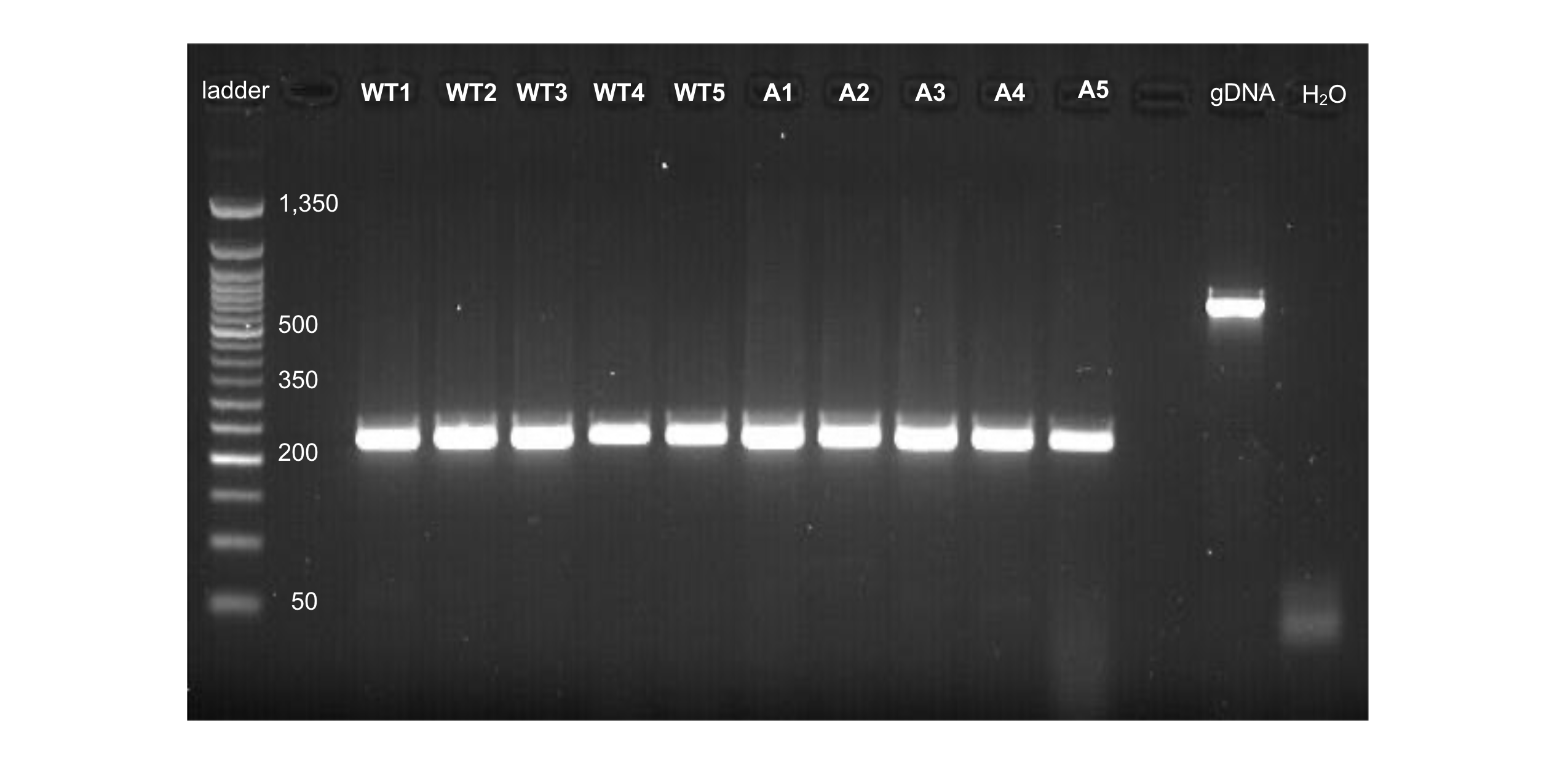

Supplement: S7 Fig — WT = control horses, A1-A5 represents individual eMD horses, WT1–5 represent controls. No alternative splicing was observed where exon 22 was excluded. (TIFF) [file pone.0341655.s007.tiff]
